# Supplementary material for: Fast microwave heating-based one-step synthesis of DNA and RNA modified gold nanoparticles
Source: Nat Commun. 2022 Feb 18;13:968. doi: 10.1038/s41467-022-28627-8 (PMC8857241; doi:10.1038/s41467-022-28627-8)
Supplement: Supplementary file 1 — Supplementary Information [file 41467_2022_28627_MOESM1_ESM.pdf]

Supplementary Information

Fast microwave heating-based one-step synthesis of DNA and RNA modified gold nanoparticles

Mengqi Huang[1†], Erhu Xiong[1†*], Yan Wang[2], Menglu Hu[1], Huahua Yue[1], Tian Tian[1], Debin Zhu[3], Hong Liu[2], and Xiaoming Zhou[1*]

[1]School of Life Sciences, South China Normal University, Guangzhou 510631, China

[2]Key Laboratory of Theoretical Chemistry of Environment Ministry of Education, School of Chemistry, South China Normal University, Guangzhou 510006, China

[3]Guangzhou Key Laboratory of Analytical Chemistry for Biomedicine, School of Chemistry, South China Normal University, Guangzhou 510006, China

[†]These authors contributed equally: Mengqi Huang, Erhu Xiong

*To whom correspondence should be addressed. E-mail: xiongeh2019@m.scnu.edu.cn or zhouxm@scnu.edu.cn

Contents

1. Materials and apparatus····························S2–S3
2. DNA damage assay····························S3–S5
3. Molecular simulations····························S5–S8
4. Supplementary Figures 1–21····························S9–S30
5. Supplementary Tables 1–3····························S31–S41

Materials and apparatus

All oligonucleotide sequences synthesized by Sangon Biotech (Shanghai, China) are listed in Supplementary Tables 2 and 3. Sodium citrate tribasic dihydrate (catalog number: A100101), Sodium phosphate dibasic anhydrous (catalog number: A501727), sodium phosphate monobasic dihydrogen (catalog number: A502805), 30% acrylamide, ammonium persulfate (APS, catalog number: A100486), N,N,N′,N′-Tetramethylethylenediamine (TEMED, catalog number: A100761), agarose (catalog number: A620014), NaCl, Tris-HCl solution (pH 7.5), ascorbic acid, magnesium chloride, 20× SSC buffer, Tween-20 (catalog number: A100777), bovine serum albumin (BSA, catalog number: A600903), n-butanol (catalog number: A501800), HEPES buffer (catalog number: E607018), 50× TAE buffer (catalog number: B548101), 5× TBE buffer (catalog number: B548102), Triton X-100 (catalog number: A110694), sucrose (catalog number: A202792), streptavidin (catalog number: A610492), DEPC-treated water, NTPs mix and deoxynucleotide (dNTP) mix were purchased from Sangon Biotech (Shanghai, China). Cetyltrimethylammonium bromide (CTAB, catalog number: C-3960-53) was purchased from Thermo Fisher Scientific. β-mercaptoethanol (ME, catalog number: 63689), sodium borohydride ($NaBH_4$, catalog number: 480886), silver nitrate ($AgNO_3$, catalog number: 204390) and $HAuCl_4$ solution (catalog number: 484385) were purchased from Sigma-Aldrich (St. Louis, MO, USA). The Bst 2.0 WarmStart DNA polymerase (catalog number: M0538L), DNase I (catalog number: M0303S), T4 endonuclease V (catalog number: M0308S), Endonuclease III (Nth) (catalog number: M0268S), T4 DNA ligase (catalog number: M0202S), and T7 RNA polymerase (catalog number: M0251L) were supplied by New England Biolabs (Beijing, China). *Streptococcus pyogenes* Cas9 protein was purchased from Bio-lifesci., Ltd. (catalog number: M20101-0500, Guangzhou, China). Premix Taq™ version 2.0 (catalog number: RR003A), 20 bp marker, DL2000 marker (catalog number: 3427A), 6× loading buffer and recombinant RNase inhibitor (RRI, catalog number: 2313A) was purchased from Takara Biotechnology Co., Ltd (Dalian, China). SARS-CoV-2 RNA standards were obtained from National Institute of Metrology (Beijing, China). The RNA Clean & Concentrator Kits was obtained from ZYMO RESEARCH (Catalog number: R1017, Beijing, China). The nitrocellulose membrane (NC) was purchased from Sartorius (Shanghai, China). The sample pad, bonding pad, bottom plate and absorbent pad were obtained by Shanghai Jieyi Biotechnology Co., Ltd. (Shanghai, China).

The absorption and fluorescence spectra were measured by SpectraMax iD5 Multimode Microplate Reader (Molecular Devices, CA, USA). Real-time fluorescence signal was recorded by a Thermal Cycler Dice™ Real Time System III (Takara, Beijing, China). Domestic microwave oven (MZC-2070M1) that used for heating was purchased from Haier company. The hydrodynamic diameters of particles were measured by a Zetasizer Nano-ZS instrument (Malvern Instruments). The particle size was measured by 200 kV Transmission Electron Microscope (JEM-2100HR, JEOL, Japan). Ultrapure water was provided by Millipore system (Milli-Q, Millipore, >18.25 MΩ·cm). The agarose gel electrophoresis and PAGE experiments were performed using equipment from Beijing Liuyi Instruments (Beijing, China). BG-gdsAUTO320 gel imaging analysis system (Beijing, China) was used for imaging analysis. The XYZ three-dimensional film and spray gold instrument used for preparing lateral flow strip was purchased from Shanghai Jinbiao Biotechnology Co., Ltd. (Shanghai, China). The concentration of nucleic acids was determined by Nanodrop 2000 (Thermo Fisher Scientific). Instrument used for PCR experiment and isothermal incubation were purchased from Eppendorf. Centrifugation of AuNPs probes was performed with Eppendorf centrifuge (5418R, Hamburg, Germany). The Raman Spectroscopy was measured using Renishaw inVia micro-spectrometer (Derbyshire, England). The CD spectrum was collected by a JASCOJ-810 spectropolarimeter (Tokyo, Japan).

DNA damage assay

Except for the DNA breakage, DNA damage also includes other different types, such as oxidation, alternation, pyrimidine dimer, and AP sites (apurinic/apyrimidinic sites, sites of base loss). In the study, we designed experiments to evaluate the possibility of two typical DNA damages (pyrimidine dimer and AP site).

First, we adopted a PCR based method[1,2] to evaluate the possibility of pyrimidine dimers. As shown in Supplementary Fig. 7a, the single-stranded DNA strands (ssDNA, 120 bp) were treated with MW-assisted heating-dry method. After quantification, the resulted DNA strands were serially diluted for PCR amplification. Finally, the PCR products were analyzed by gel electrophoresis. Meanwhile, for comparison, the PCR products amplified by the same concentration of DNA strands without MW-assisted heating-dry treatment were also analyzed by gel electrophoresis. If the pyrimidine dimers are generated on the DNA strands during the MW-assisted heating-dry process, they will hinder the polymerase extension reaction, resulting in reduced or no PCR products generation. While the PCR will proceed normally in the absence of

pyrimidine dimers. As shown in Supplementary Fig. 7b, regardless of MW-assisted heating-dry treatment, no significant differences were found after PCR amplification with the same concentration of DNA strands, even at a relatively low DNA concentration (1 fM), indicating that the MW-assisted heating-dry treatment did not generate potential pyrimidine dimers on the DNA strands.

Next, we detected the AP sites (apurinic/apyrimidinic sites, sites of base loss) by repair endonucleases. Combination of T4 endonuclease V, Endonuclease III (Nth), glycosylase and AP endonuclease activity can be used for recognizing and cleaving the AP sites to generate the DNA strand breaks[3]. According to the design, the modified bases would be removed by the enzyme from the deoxyribose moiety and then the DNA backbone at the generated AP sites would be cleaved.

We first treated the short-chain ssDNA and long-chain EGFP plasmid DNA with MW-assisted heating-dry method, and then added them in the endonuclease reaction system, respectively, and finally analyzed the products by gel electrophoresis (Supplementary Fig. 8a). As shown in Supplementary Fig. 8b, no matter whether the MW-assisted heating-dry treatment was performed, no cleaved DNA fragments were observed, indicating that the MW-assisted heating-dry process did not cause the generation of AP sites. Additionally, because T4 endonuclease V can also recognize and cleave the 5'-end glycosidic bond of the pyrimidine dimer, while its endonuclease activity can cleave the phosphodiester bond at the AP site, leading to DNA breaks. Therefore, these experimental results once again prove that the MW-assisted heating-dry process does no cause the formation of pyrimidine dimers.

Experimental conditions used for evaluating DNA damage. (i) PCR method for the evaluation of pyrimidine dimers. First, the ssDNA strands were heated with MW-assisted heating-dry method. After quantification, the resulted DNA strands were serially diluted for PCR amplification. 50 μL PCR solution (25 μL 2× Taq mix, 20 μL ddH_2O, 2 μL of 10 μM forward and reverse primers, 1 μL template with different concentrations) was performed at 95 °C for 5 min, followed by 30 cycles of amplification at 95 °C for 30 s, 57 °C for 30 s, 72 °C for 30 s, finally incubated at 72 °C for 10 min. Subsequently, the PCR products were analyzed by 10% native PAGE. (ii) Repair endonuclease method. First, the short-chain ssDNA and long-chain EGFP plasmid were treated by the MW-assisted heating-dry method, respectively. Next, added the resulted nucleic acids in the endonuclease reaction solution including 0.1 μg/μL BSA, 0.5 U/μL T4 endonuclease V and

Endonuclease III (Nth), and incubated at 37 °C for 30 min. Finally, the resulted solutions were analyzed by 10% native PAGE and 0.8% agarose gel, respectively.

Molecular simulations

Strategy for models building. The molecular models of different poly (T)-DNA sequences were built with the aid of the Nucleic Acid Builder (NAB), which is a high-level language that facilitates manipulations of macromolecules and their fragments. The NAB is included as part of the AmberTools 21 software package. We selected a certain dimension of face-centered cubic (FCC) AuNPs surface (Au (100)) to represent AuNPs. All the molecular models containing Au (100) and poly (T)-DNA sequences are constructed using PackMOL software package[4].

Molecular dynamics simulations. In this study, the Parmbsc1 force field was chosen for the poly (T)-DNA molecules[5]. The FCC metal parameters from the previous report[6] combined in the interface force field was selected for the Au (100) surface. All the MD simulations were performed with the aid of AMBER 20 software package[7]. The topology files that are essential for MD simulations were prepared by the LEAP module of AMBER 20 software package[7]. Additionally, the periodic boundary condition was used in the simulations.

The simulation strategies used to MD simulations on the models are as follows.

Minimization. Based on the models obtained by PackMOL software, energy minimization was conducted to obtain a lower-energy starting conformations, which will be used as the starting points for the subsequent MD simulations. Practically 8000 steps of steepest descent and 12000 steps of conjugated gradient minimizations were performed.

Heating. The models were then heated under NVT conditions from 0 to 378 K for 1 ns, with the Langevin Thermostat applied. The force constant for the harmonic restraint was set to be 10.0 kcal mol^{-1} $Å^{-2}$.

Equilibration. After heating, in order to guarantee the quality of the model structures, we employed equilibration simulations to further optimize the model structures. The equilibration simulations were performed for 100 ns in total. During the simulations, a restraint, which was gradually reduced from 10 to 0.05 kcal mol^{-1} $Å^{-2}$, was applied to the model structure. The restraint is of the form $k(\Delta x)^2$, where k is the restraint value and Δx is the difference between one of the Cartesian coordinates of a restrained atom and its reference position. The equilibration simulations were carried out under NPT conditions.

Production run. All the model structures were finally simulated for 100 ns under NPT with periodic boundary conditions. The relaxation time for barostat bath was set to 2.0 ps. The Langevin Thermostat and the Berendesen barostat were applied to preserve the constant temperature and pressure. The particle-mesh Ewald (PME) method[8] is employed to handle the long-range electrostatic interactions. We picked up the value of 10.0 Å as the cut-off value to deal with the short-range interactions. The time step used in all the MD simulations is set to be 2 fs.

Binding free energy calculations. The molecular mechanics/generalized born surface area (MM/GBSA) method is designed to calculate the binding free energy, which aims to estimate the binding free energy between the ligand and its receptor[9]. The binding free energy is often used to characterize the binding strength between a receptor and a ligand molecule. The binding free energy (ΔG_{bind}) in MM/GBSA between a ligand (L) and a receptor (R) to form a complex RL was calculated as:

$$\Delta G_{bind} = G_{complex} - \left(G_{receptor} + G_{ligand}\right) \qquad (1)$$

The ΔG_{bind} can be decomposed into contributions of different interactions and expressed as[10]:

$$\Delta G = \Delta H - T\Delta S = \Delta E_{MM} + \Delta G_{sol} - T\Delta S \qquad (2)$$

$$\Delta E_{MM} = \Delta E_{int} + \Delta E_{ele} + \Delta E_{vdw} \qquad (3)$$

$$\Delta G_{sol} = \Delta G_{GB} + \Delta G_{SA} \qquad (4)$$

$$\Delta G_{SA} = \gamma \cdot SASA + b \qquad (5)$$

In the Equation (1), the ΔE_{MM} represents the molecular mechanics component. This component was determined in gas phase. ΔG_{sol} is the stabilization energy. This term is caused by solvation. $T\Delta S$ represents the vibrational entropy term. The ΔE_{MM} term is a sum of three terms, as shown in the Equation (1). ΔE_{int}, ΔE_{ele}, and ΔE_{vdw} are internal, Coulomb and van der Waals interaction terms, respectively. The Equation (2) gives the solvation energy, ΔG_{sol}. As shown in this equation, the solvation energy can be divided into two terms, the electrostatic solvation free energy (ΔG_{GB}) and the nonpolar solvation free energy (ΔG_{SA}). The ΔG_{GB} term is obtained by the Generalized Born (GB) method. The ΔG_{SA} term can be proportional to the molecular solvent accessible surface area (SASA) method[11,12]. In the Equation (5), γ is the surface tension constant

and b is a correction constant (γ = 0.00542 kcal mol^{-1} Å^{-2} and b = 0.92 kcal mol^{-1} in the AMBER package). Based on the 25 ns ASMD simulation of each system, binding free energies were calculated. The conformational entropy was taken into consideration and was calculated by using the normal mode (nmode) analysis[10].

In order to clearly show the binding free energies between poly (T)-tagged DNA sequences and the AuNPs surface during the adsorption process, the total binding free energy was decomposed into contributions from individual bases ($i = 2, 3, \cdots, 11, 16$):

$$G_{bind} = \sum_{i=2}^{11,16} G_{bind}^{i} = \sum_{i=2}^{11,16} \sum_{j \neq i}^{11,16} G_{bind}^{i,j} \qquad (6)$$

where G_{bind}^{i} are the pre-residue contributions and $G_{bind}^{i,j}$ are the residue-pairwise interaction contributions. All the calculations of binding free energy were performed in the MMPBSA.py module[13] of AMBER 20.

In the molecular simulations, we employed two different poly (T)-tagged DNA sequences (5'-poly (T_5)-CACTAG-3' and 5'-poly (T_{10})-CACTAG-3') as the template to construct the molecular models. The molecular dynamics (MD), adaptive steered molecular dynamics (ASMD) and binding free energy calculations are adopted here to clarify the above-mentioned concerns.

First, the all-atom molecular model of these two poly (T)-tagged DNA sequences were constructed with the aid of Nucleic Acid Builder (NAB). Then these two DNA models were optimized by means of MD simulations under the same conditions as used in the experiments. The face-centered cubic (FCC) AuNPs surface (Au (100)), with the size of $155.0 \times 155.0 \times 30.0$ Å^3, was selected to represent the AuNPs. The model of Au (100) surface was built in the LEAP module of AmberTools 21. Here, the ASMD method was employed to simulate the adsorption process which occurred between different poly (T)-tagged DNA sequences and AuNPs. As shown in Supplementary Fig. 18a, with the aid of PackMOL software[4], the models of two poly (T)-tagged DNA strands were placed far above the Au (100) surface.

In order to clearly describe the interaction between the two poly (T)-tagged DNA strands and Au (100) surface during the adsorption process, the binding free energy calculations were employed. As shown in Supplementary Fig. 18b, the binding free energy between poly (T_{10})-tagged DNA strand and Au (100) surface are much higher than that of poly (T_5)-tagged DNA strand, indicating that with the same affinity sequence, the poly (T_{10})-tagged DNA strand is more

inclined to be adsorbed on the AuNPs surface than the poly (T_5)-tagged DNA strand, and more T bases may provide higher driving force to attach on the AuNPs surface.

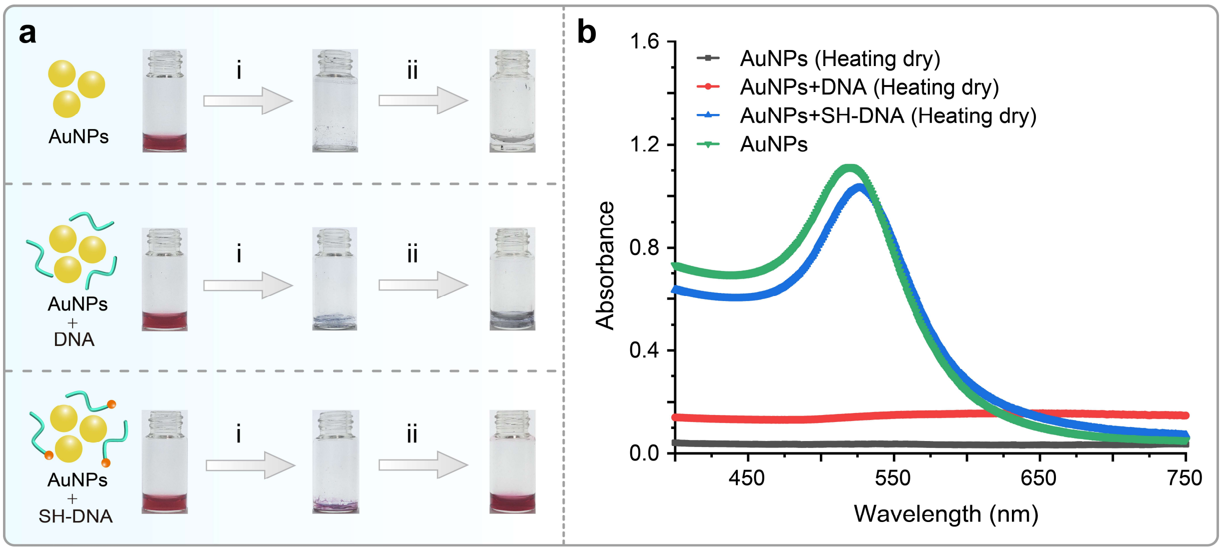

Supplementary Fig. 1 Fast construction of thiolated DNA/RNA-AuNP conjugates based on MW-assisted heating-dry method. (a) Photographs of AuNPs before and after MW-assisted heating-dry process. The bare AuNPs and DNA/RNA sequence mixed AuNPs aggregated while SH-DNA/AuNPs retained monodispersed and seems red after heating dry and resuspension. i: MW-assisted heating dry; ii: resuspending with water or buffer. **(b)** Characterization of the SH-DNA-AuNP conjugates based on MW-assisted heating-dry labeling method using UV-vis absorption spectroscopy. The absorption peak of AuNPs with SH-DNA exhibits a 6 nm red-shift, suggesting that there are DNA attached on the AuNPs surface.

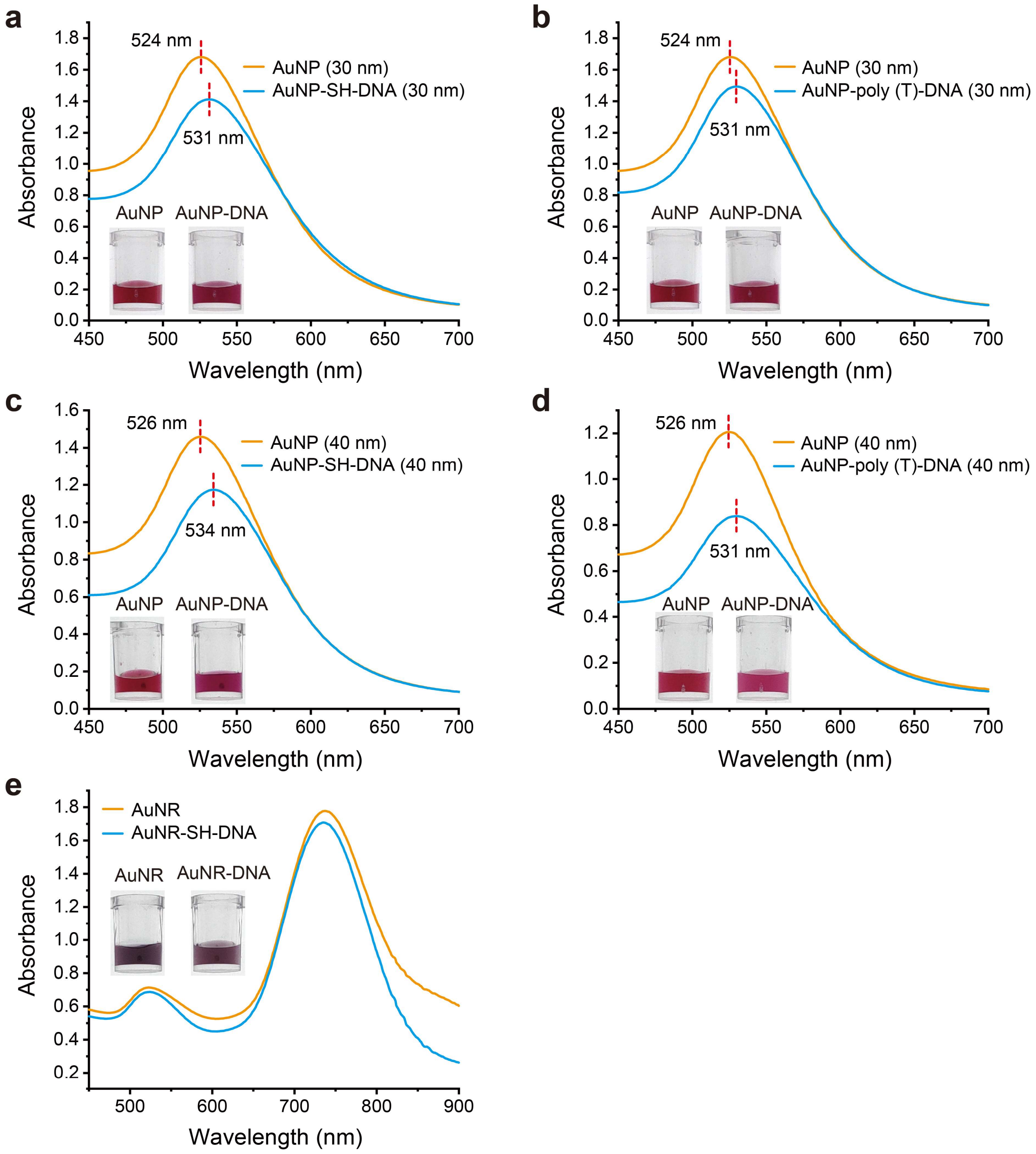

Supplementary Fig. 2 MW-assisted heating-dry labeling of thiolated and non-thiolated DNA with larger-sized AuNPs and AuNRs. (a) UV-vis absorption spectroscopy and photographs of 30 nm AuNPs before and after MW-assisted heating-dry labeling of SH-DNA. **(b)** UV-vis absorption spectroscopy and photographs of 30 nm AuNPs before and after MW-assisted heating-dry labeling of poly (T)-tagged DNA. Compared to the bare AuNPs, a 7 nm red-shift is observed, suggesting that SH-DNA and poly (T)-tagged DNA successfully attached on the 30 nm AuNPs surface. **(c)** UV-vis absorption spectroscopy and photographs of 40 nm AuNPs before and after

MW-assisted heating-dry labeling of SH-DNA. **(d)** UV-vis absorption spectroscopy and photographs of 40 nm AuNPs before and after MW-assisted heating-dry labeling of poly (T)-tagged DNA. Compared to the bare AuNPs, 8 and 5 nm red-shift are observed, suggesting that SH-DNA and poly (T)-tagged DNA successfully attached on the 40 nm AuNPs surface. **(e)** UV-vis absorption spectroscopy and photographs of AuNRs before and after MW-assisted heating-dry labeling of SH-DNA.

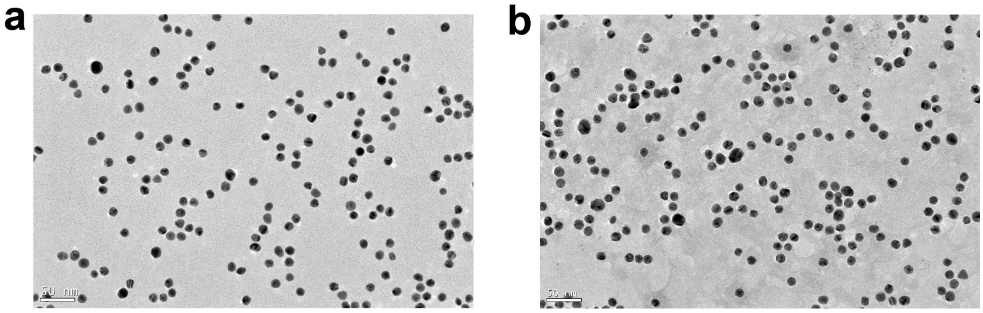

Supplementary Fig. 3 Transmission electron microscope analysis. (a) Bare AuNPs, **(b)** Poly (T)-tagged DNA-AuNP conjugates.

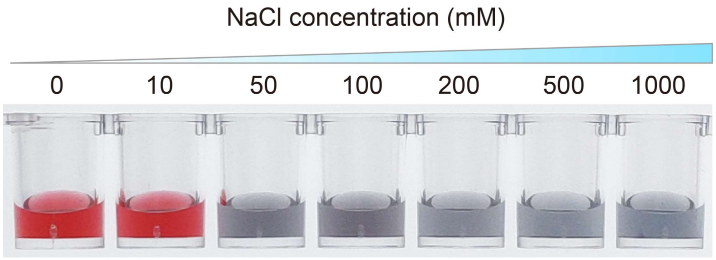

Supplementary Fig. 4 Salt stability of the bare AuNPs.

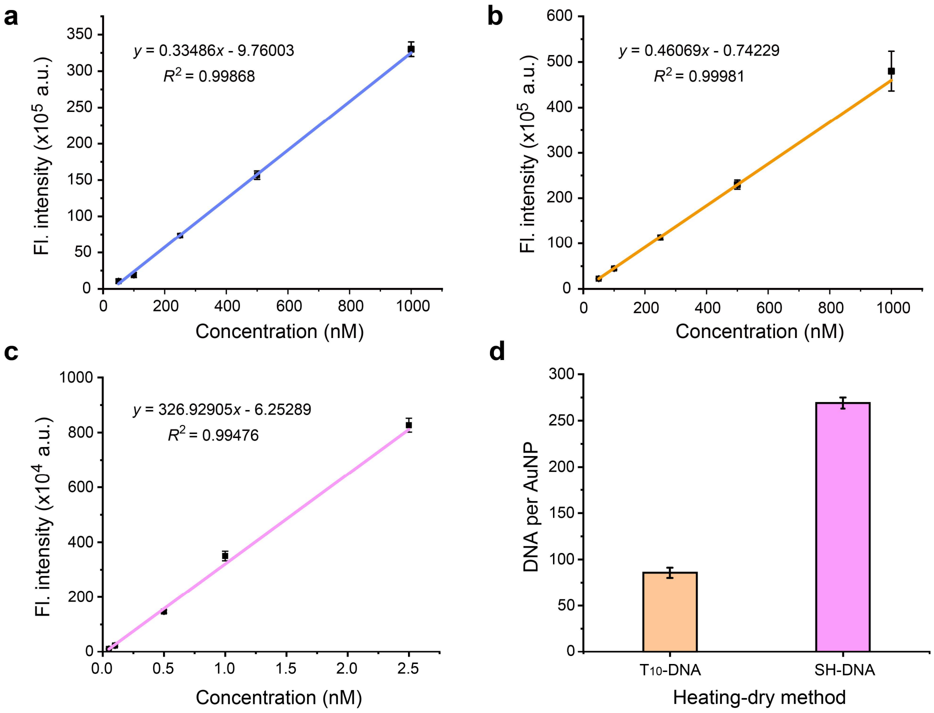

Supplementary Fig. 5 Analysis the numbers of poly (T)-tagged DNA probes and SH-DNA probes attached to each AuNP. (a) The fluorescence standard curve of FAM-labelled poly (A)-tagged DNA probe. **(b)** The fluorescence standard curve of FAM-labelled poly (T)-tagged DNA probe. **(c)** The fluorescence standard curve of FAM-labelled SH-DNA probe. **(d)** The measured number of DNA probes attached to each AuNP. The DNA-AuNP conjugates were constructed with poly (T_{10})-tagged DNA and SH-DNA using MW-assisted heating-dry method. Error bars represent three different individual experiments. The number of SH-DNA attached to each AuNP is 269, which is about three times as much as poly (T)-tagged DNA. a.u., arbitrary units. Error bars = standard deviation (n = 3).

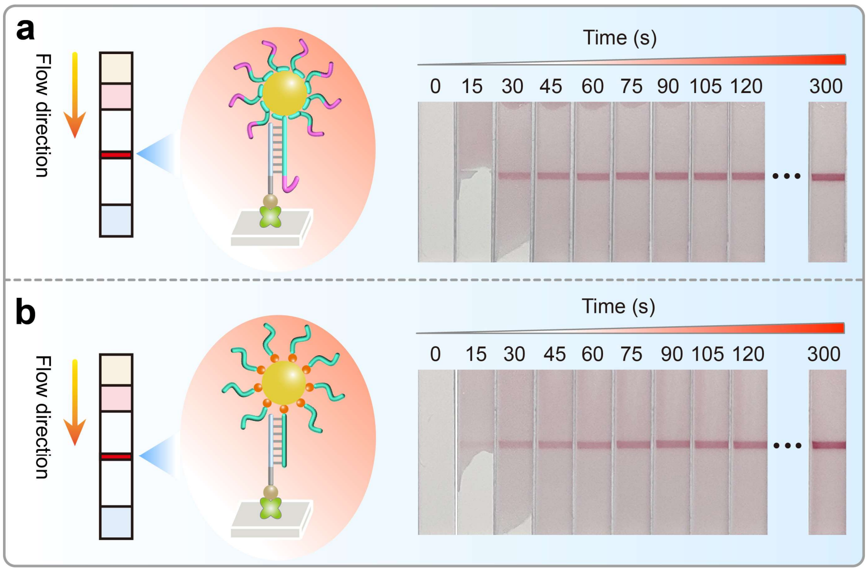

Supplementary Fig. 6 Evaluation of hybridization ability of non-thiolated and thiolated DNA-AuNP conjugates using test strip assay. Non-thiolated DNA-AuNPs conjugates were constructed by MW-assisted heating-dry method and SH-DNA-AuNP conjugates were constructed by freeze-thaw method. The streptavidin-biotinylated DNA probes complementary to DNA-AuNP conjugates were pre-embedded in the test strip. For test strip assay, 10 μL resuspended DNA-AuNP conjugates were firstly mixed with 10 μL running buffer and then added to the sample pad of the test strip device. Then, 100 μL running buffer was added on the sample pad to wash the test strip, and the test strip was photographed per 15 s. Photographs showing that the **(a)** non-thiolated as well as **(b)** SH-DNA-AuNP conjugates could be efficiently captured by the biotinylated DNA probes on test strip.

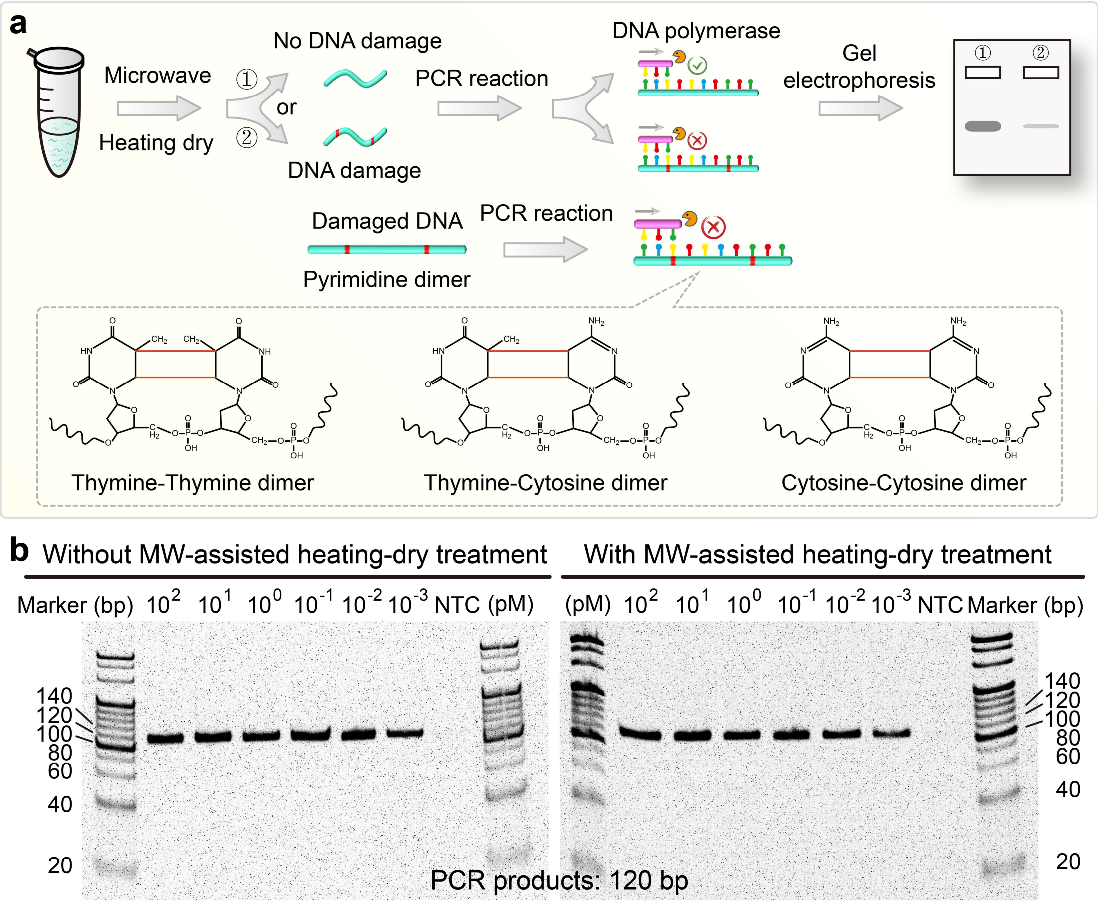

Supplementary Fig. 7 Evaluation of potential pyrimidine dimers generation under MW-assisted heating-dry treatment. (a) Scheme of the evaluation method. **(b)** Gel electrophoresis analysis of PCR products amplified by different concentrations of DNA strand with or without MW-assisted heating-dry treatment.

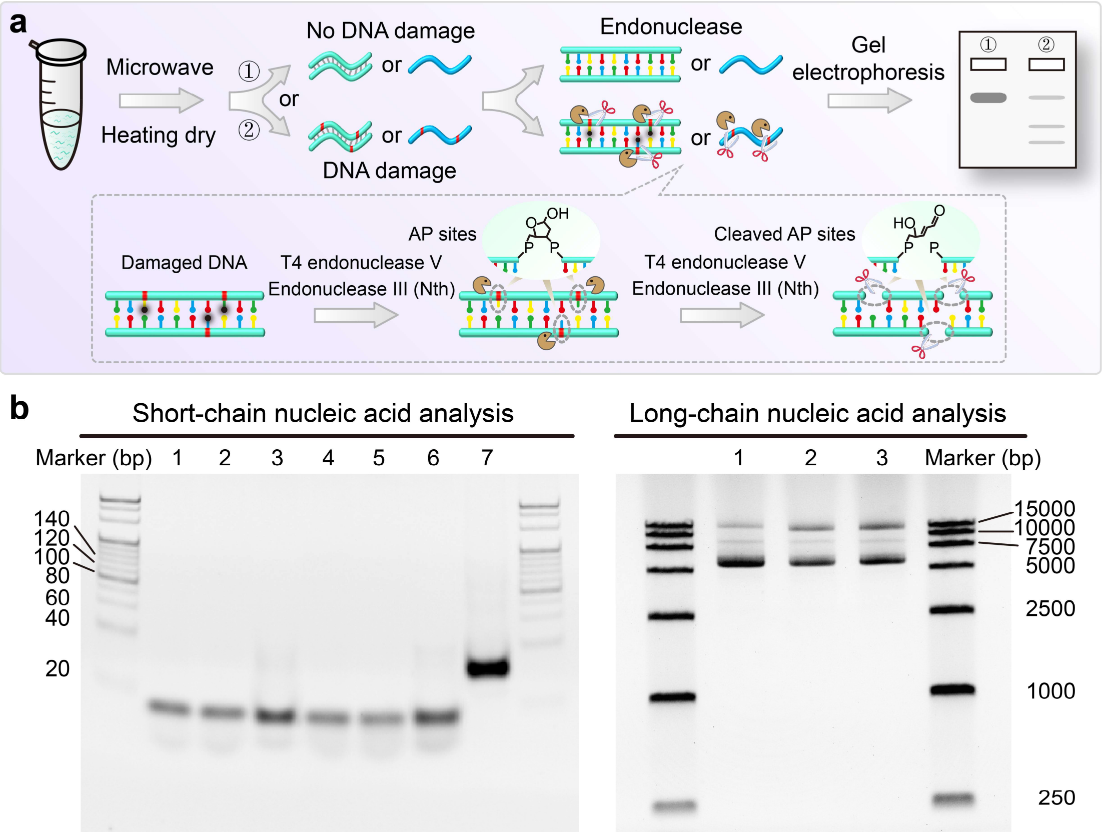

Supplementary Fig. 8 Evaluation of potential AP sites generation under MW-assisted heating-dry treatment. (a) Scheme of the evaluation method. **(b)** Gel electrophoresis analysis of short-chain ssDNA and long-chain EGFP plasmid DNA with or without MW-assisted heating-dry treatment in the absence/present of endonuclease. Left panel: lanes 1 and 4, ssDNA and C-ssDNA (complementary to ssDNA) without MW-assisted heating-dry treatment; lanes 2 and 5, ssDNA and C-ssDNA with MW-assisted heating-dry treatment; lanes 3 and 6, ssDNA and C-ssDNA with MW-assisted heating-dry and endonuclease treatment; lane 7, ssDNA/C-ssDNA duplex with MW-assisted heating-dry and endonuclease treatment. Right panel: lane 1, EFGP plasmid DNA without MW-assisted heating-dry treatment; lane 2, EFGP plasmid DNA with MW-assisted heating-dry treatment; lane 3, EFGP plasmid DNA with MW-assisted heating-dry and endonuclease treatment.

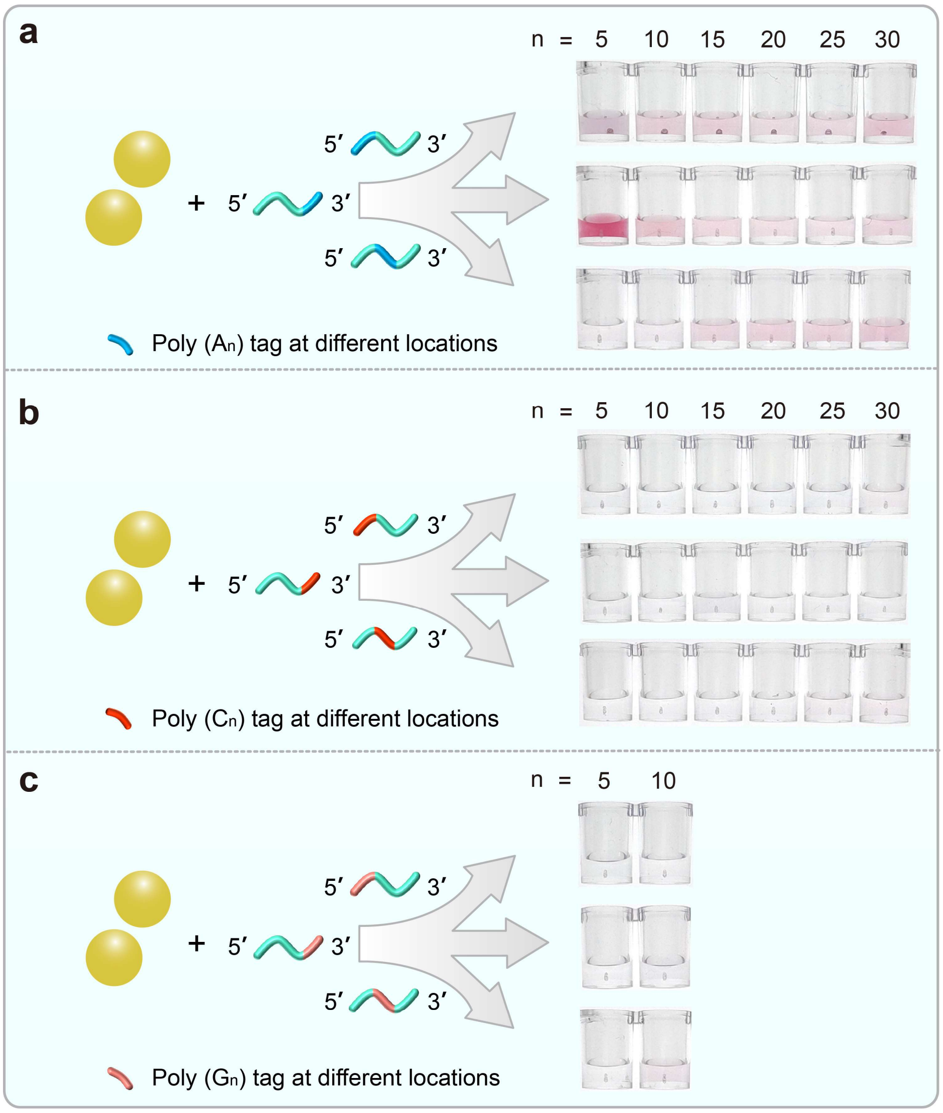

Supplementary Fig. 9 MW-assisted heating-dry labeling of poly (A/C/G)-tagged DNA. (a) Photographs showing the labeling results of different lengths of poly (A)-tagged DNA at 5′-terminal, 3′-terminal, and in the middle region. The length of poly (A) tag is 5, 10, 15, 20, 25, and 30, respectively. **(b)** Photographs showing the labeling results of different lengths of poly (C)-tagged DNA at 5′-terminal, 3′-terminal, and in the middle region. The length of poly (A) tag is 5, 10, 15, 20, 25, and 30, respectively. **(c)** Photographs showing the labeling results of different lengths of poly (G)-tagged DNA at 5′-terminal, 3′-terminal, and in the middle region. The length of poly (G) tag is 5 and 10. Longer poly (G) is hard to synthesized. An inefficient and failed labeling will lead to the AuNPs aggregation.

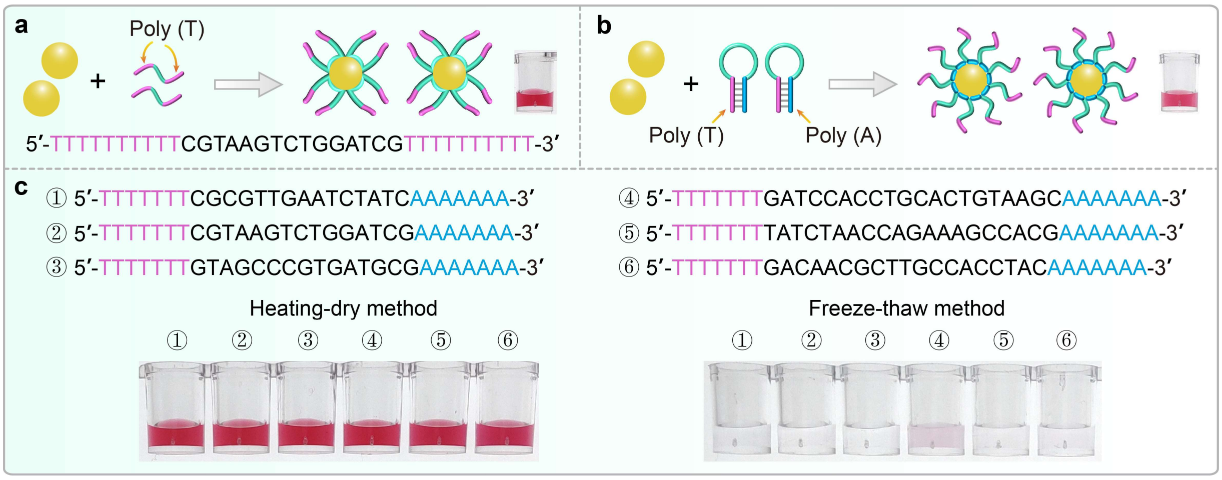

Supplementary Fig. 10 Labeling of structured DNA. (a) MW-assisted heating-dry method for labeling of DNA with poly (T) tag located at both 5′- and 3′-terminal. **(b)** MW-assisted heating-dry method for labeling of hairpin DNA sequence with shielded poly (T) tag located in the stem region. **(c)** Photographs showing the labeling results of six hairpin DNA sequences with shielded poly (T) tag located in the stem region using MW-assisted heating-dry method and freeze-thaw method, respectively.

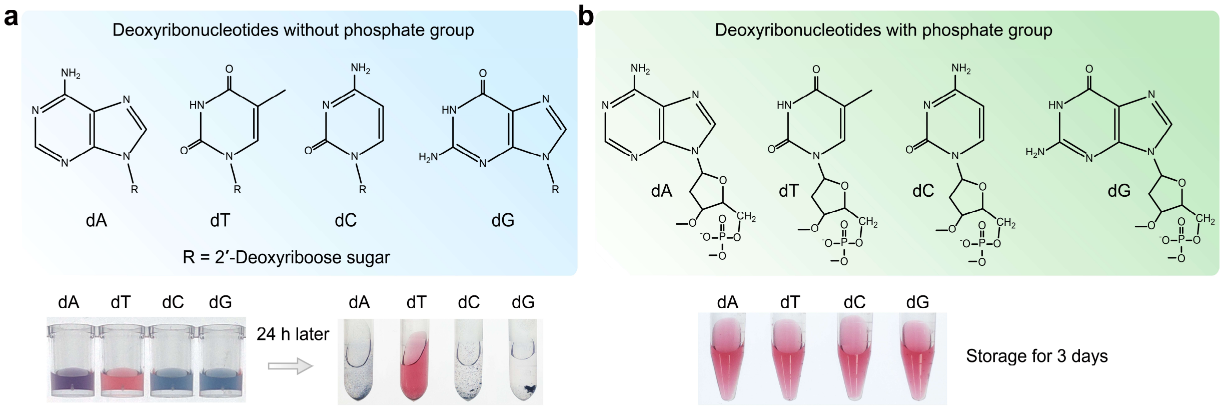

Supplementary Fig. 11 Colorimetric evaluation of deoxyribonucleotides-Au binding affinity. (a) Photographs showing the binding results of phosphate group-free dA, dT, dC, and dG. 1 μL 5 mM phosphate group-free dA, dT, dC, and dG were mixed with 1 mL 6.4 nM AuNPs solution, respectively. Binding of deoxyribonucleoside to AuNPs resulted in the aggregations of AuNPs due to the replacement of citrate ion, which is a stabilizer of AuNPs. The results showed that phosphate group-free dA, dC, and dG can efficiently bind to AuNPs while dT cannot. **(b)** Photographs showing the binding results of phosphate group-contained dA, dT, dC, and dG. 1 μL 5 mM dA, dT, dC, and dG were mixed with 1 mL 6.4 nM AuNPs solution, respectively. Binding of deoxyribonucleotides to AuNPs did not result in the AuNPs aggregations because the surface charge is not replaced.

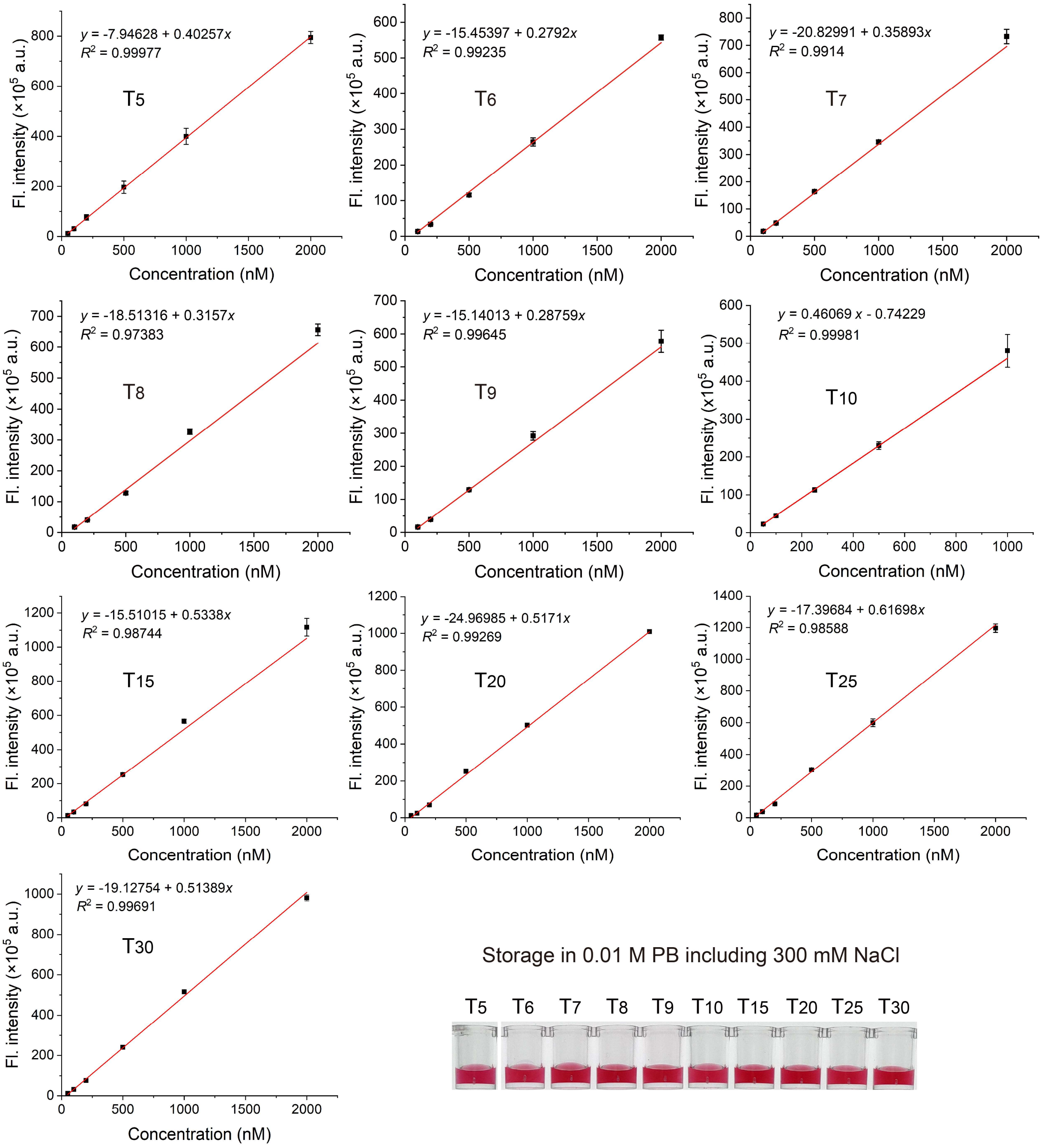

Supplementary Fig. 12 The fluorescence standard curves of FAM-labelled Poly (T_n)-Poly (C_5) DNA probes (n = 5, 6, 7, 8, 9, 10, 15, 20, 25, and 30, respectively). Photographs showing that all Poly (T_n)-Poly (C_5) DNA probes could be efficiently attached on AuNPs since the AuNPs remains monodisperse and seems red after labeling and resuspending in 0.01 M PB including 300 mM NaCl. a.u., arbitrary units. Error bars = standard deviation (n = 3).

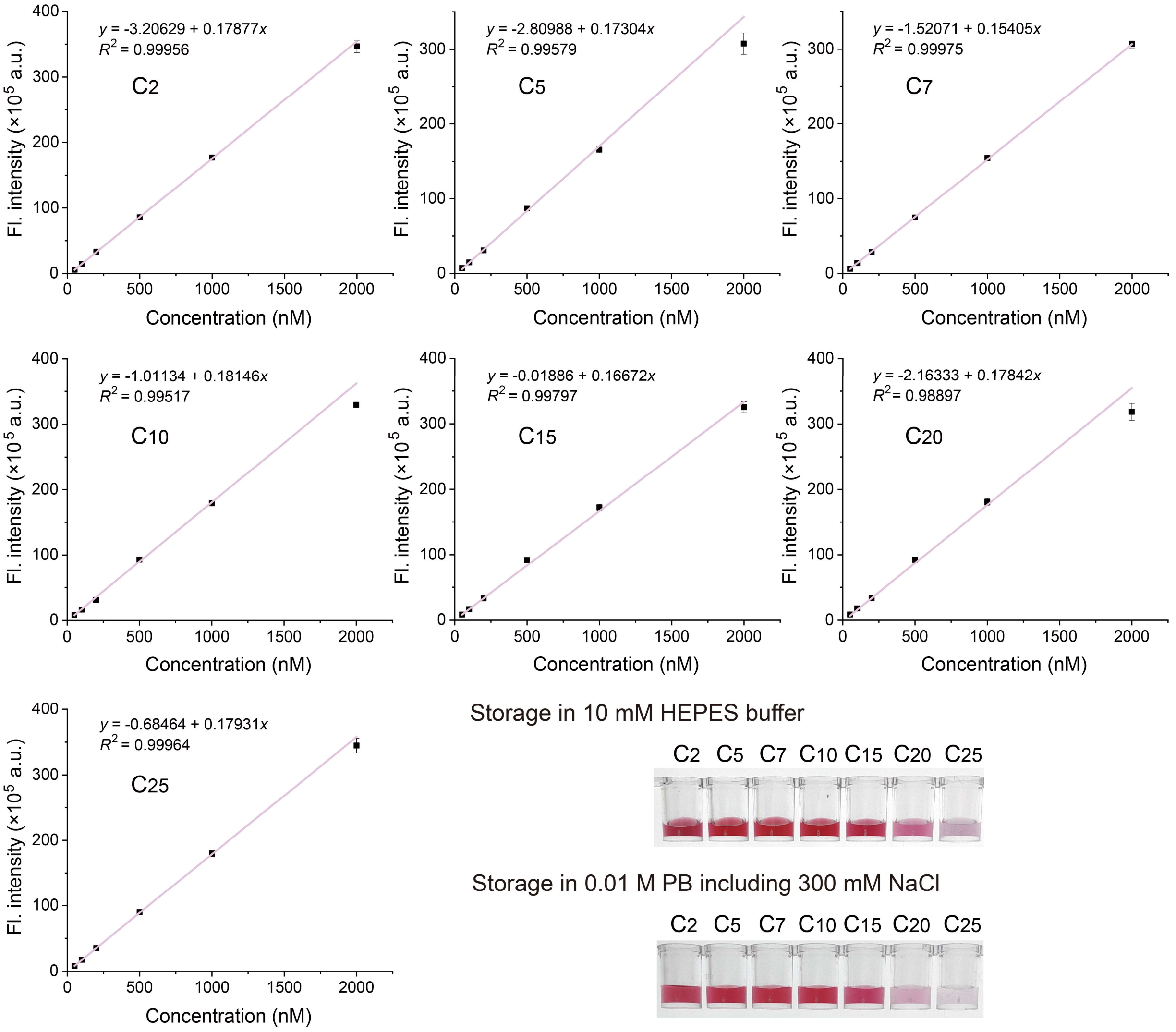

Supplementary Fig. 13 The fluorescence standard curves of FAM-labelled Poly (T_{10})-Poly (C_n) DNA probe (n = 2, 5, 7, 10, 15, 20, and 25, respectively). Photographs showing the labeling results of these Poly (T_{10})-Poly (C_n) DNA. The DNA-AuNP conjugates were resuspended in 10 mM HEPES buffer and 0.01 M PB including 300 mM NaCl. When n is over than 15, the DNA-AuNP aggregated after MW-assisted heating-drying since the number of Poly (T_{10})-Poly (C_n) DNA probe attached on each AuNP is not enough to stabilize the AuNPs. a.u., arbitrary units. Error bars = standard deviation (n = 3).

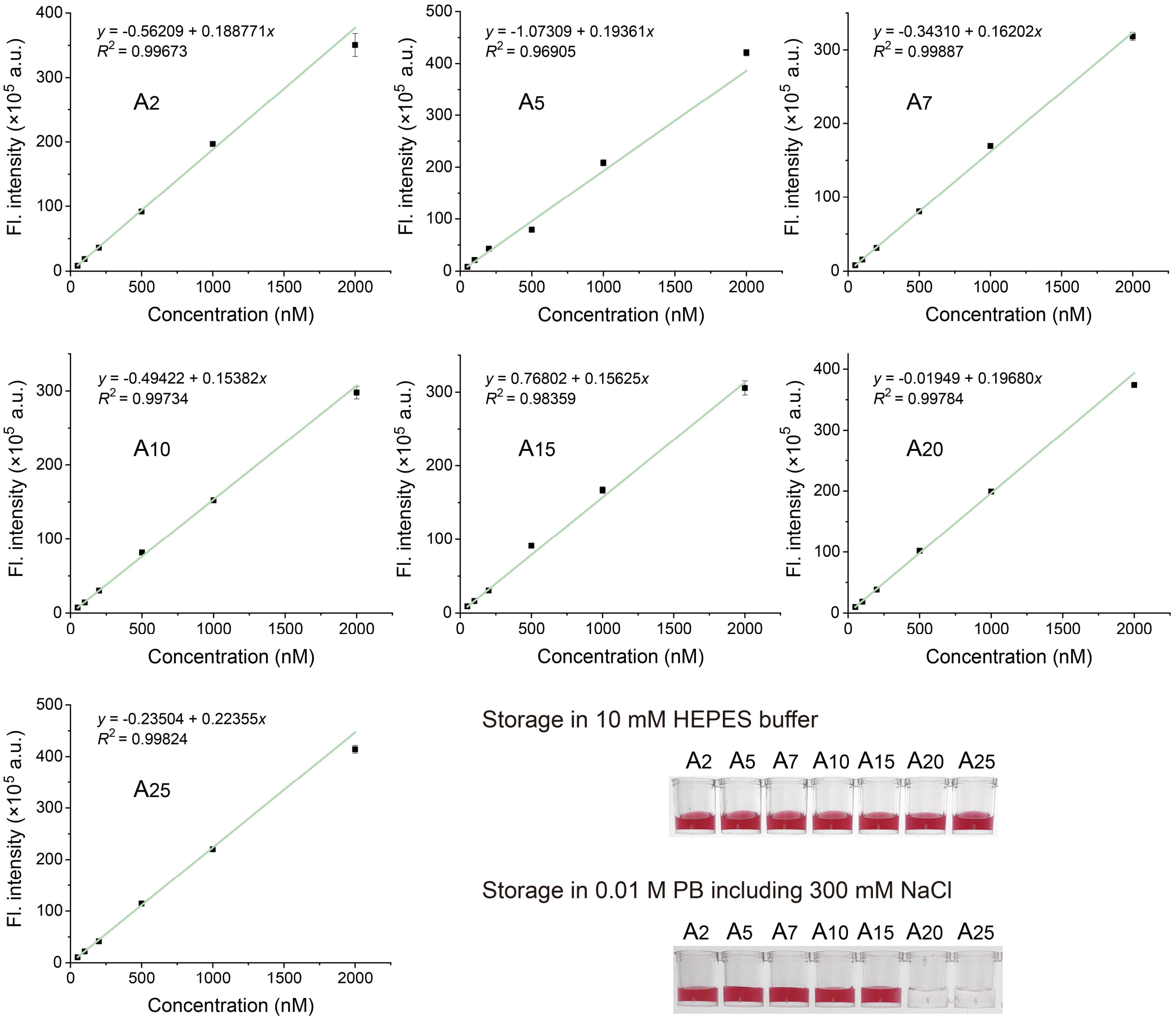

Supplementary Fig. 14 The fluorescence standard curves of FAM-labelled Poly (T_{10})-Poly (An) DNA probe (n = 2, 5, 7, 10, 15, 20, and 25, respectively). Photographs showing the labeling results of these Poly (T_{10})-Poly (A_n) DNA. The DNA-AuNP conjugates were resuspended in 10 mM HEPES buffer and 0.01 M PB including 300 mM NaCl. When n is over than 15, the DNA-AuNP remains monodisperse and seems red in 10 mM HEPES buffer while aggregated in 0.01 M PB including 300 mM NaCl since the number of Poly (T_{10})-Poly (A_n) DNA probe attached on each AuNP is not enough to stabilize the AuNP in 0.01 M PB including 300 mM NaCl. a.u., arbitrary units. Error bars = standard deviation (n = 3).

① 5′-Poly (A_{10}/T_{10}/C_{10}/G_{10})-TATCTAACCAGAAAGCCACG-3′

② 5′-Poly (A_{10}/T_{10}/C_{10}/G_{10})-GACAACGCTTGCCACCTAC-3′

③ 5′-Poly (A_{10}/T_{10}/C_{10}/G_{10})-GATCCACCTGCACTGTAAGC-3′

④ 5′-Poly (A_{10}/T_{10}/C_{10}/G_{10})-CGCGTTGAATCTATC-3′

⑤ 5′-Poly (A_{10}/T_{10}/C_{10}/G_{10})-CGTAAGTCTGGATCG-3′

⑥ 5′-Poly (A_{10}/T_{10}/C_{10}/G_{10})-GTAGCCCGTGATGCG-3′

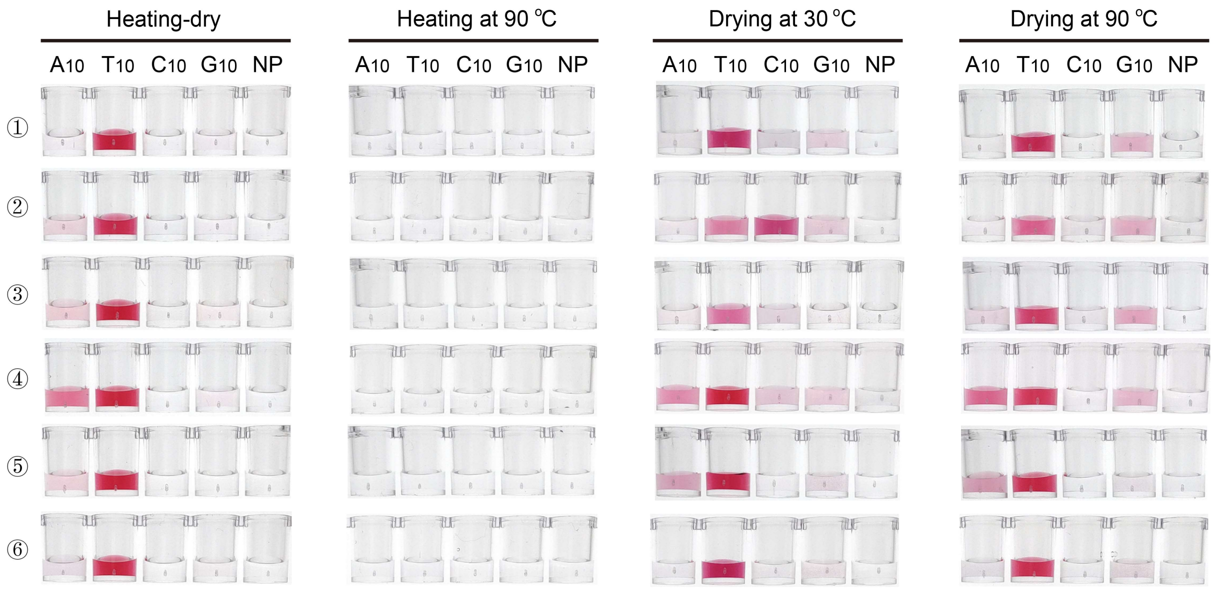

Supplementary Fig. 15 Photographs showing the labeling results of six poly (A/T/C/G)-tagged DNAs using heating-dry, or heating, or drying method. The six poly (A/T/C/G)-tagged DNA sequences are shown on the top panel. Heating-drying method was performed as described. For heating method at 90 °C, the same mixture of AuNPs and DNA probes as heating-drying method was heated at 90 °C for 1 h in a sealed tube. For drying method at 30 and 90 °C, the same mixtures of AuNPs and DNA probes as heating-drying method were dried in an air-dry status at 30 °C for 7 h and at 90 °C in an oven-dry status for 1 h, respectively. Photographs showing that all the poly (T)-tagged DNAs exhibited efficient labeling with MW-assisted heating-dry method and failed labeling with heating at 90 °C. For drying method at 30 and 90 °C, the poly (T)-tagged DNA showed decreased and uniform labeling efficiency. While with all the four labeling methods, both the poly (A/C/G)-tagged DNA and non-poly base DNA (NP) exhibited failed labeling.

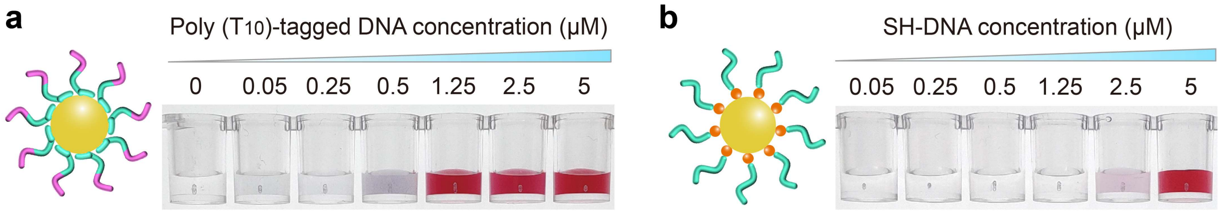

Supplementary Fig. 16 Test of the critical DNA concentration required for the MW-assisted heating-dry labeling. (a) Poly (T_{10})-tagged DNA, **(b)** SH-DNA.

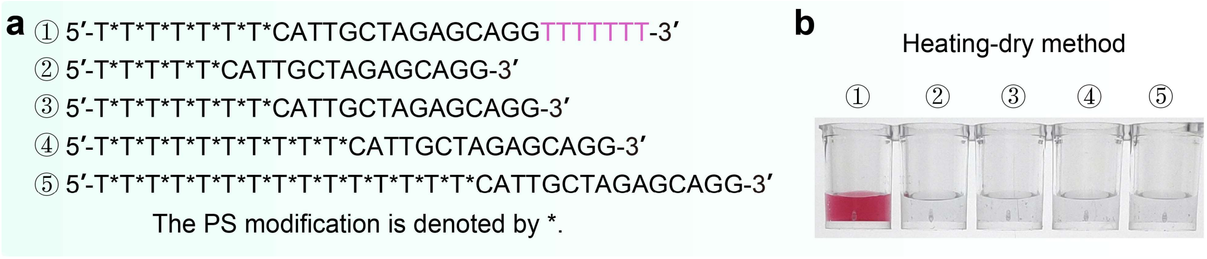

Supplementary Fig. 17 MW-assisted heating-dry method for labeling of phosphorothioate (PS)-modified DNA sequence. (a) The detail sequence of PS-modified poly (T)-tagged DNA probes. **(b)** The labeling results of these DNA probes using MW-assisted heating-dry method.

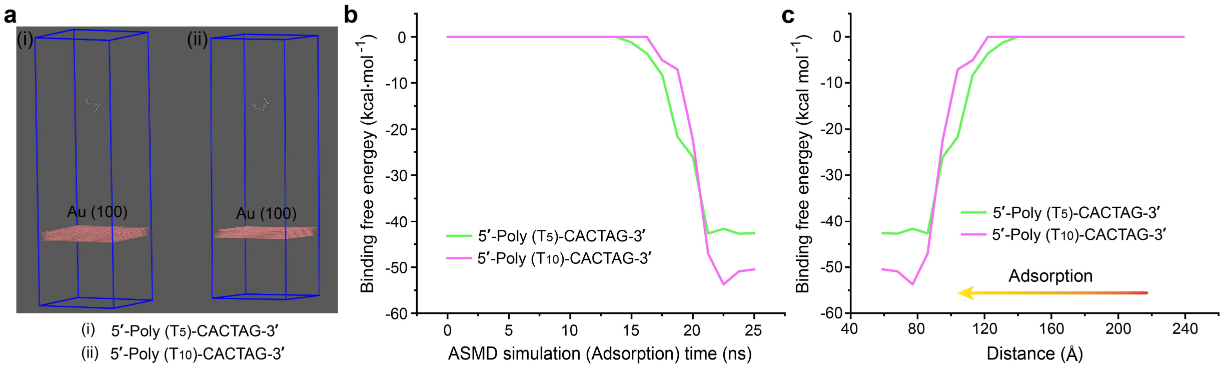

Supplementary Fig. 18 Molecular simulations of DNA strands adsorption on AuNPs with different numbers of T bases. (a) The initial structures used in the ASMD simulation with the dimension of $155.0 \times 155.0 \times 500.0$ Å^3 **(b)** The binding free energies of poly (T_5)-CACTAG and poly (T_{10})-CACTAG strands as function of adsorption time. **(c)** The binding free energies of poly (T_5)-CACTAG and poly (T_{10})-CACTAG strands as function of distance.

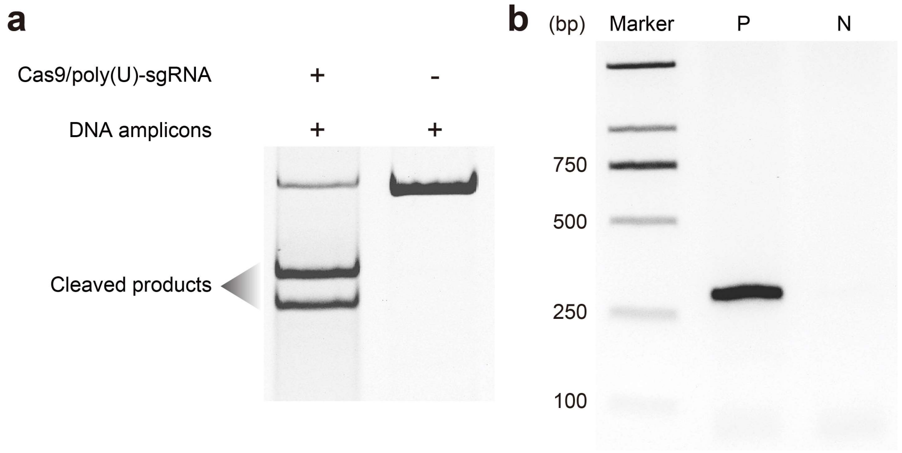

Supplementary Fig. 19 Gel electrophoretic analysis. (a) Cleavage ability evaluation using designed poly (U)-tagged sgRNA and Cas9 protein. **(b)** Gel electrophoretic analysis of the PCR products of *B646L* (VP72) gene. P and N stand for positive and negative samples, respectively.

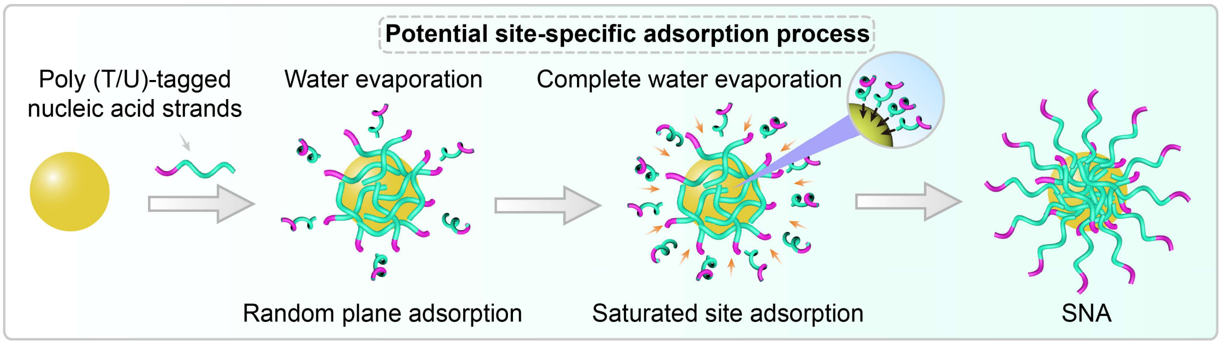

Supplementary Fig. 20 The potential site-specific adsorption process in the MW-assisted heating-dry method.

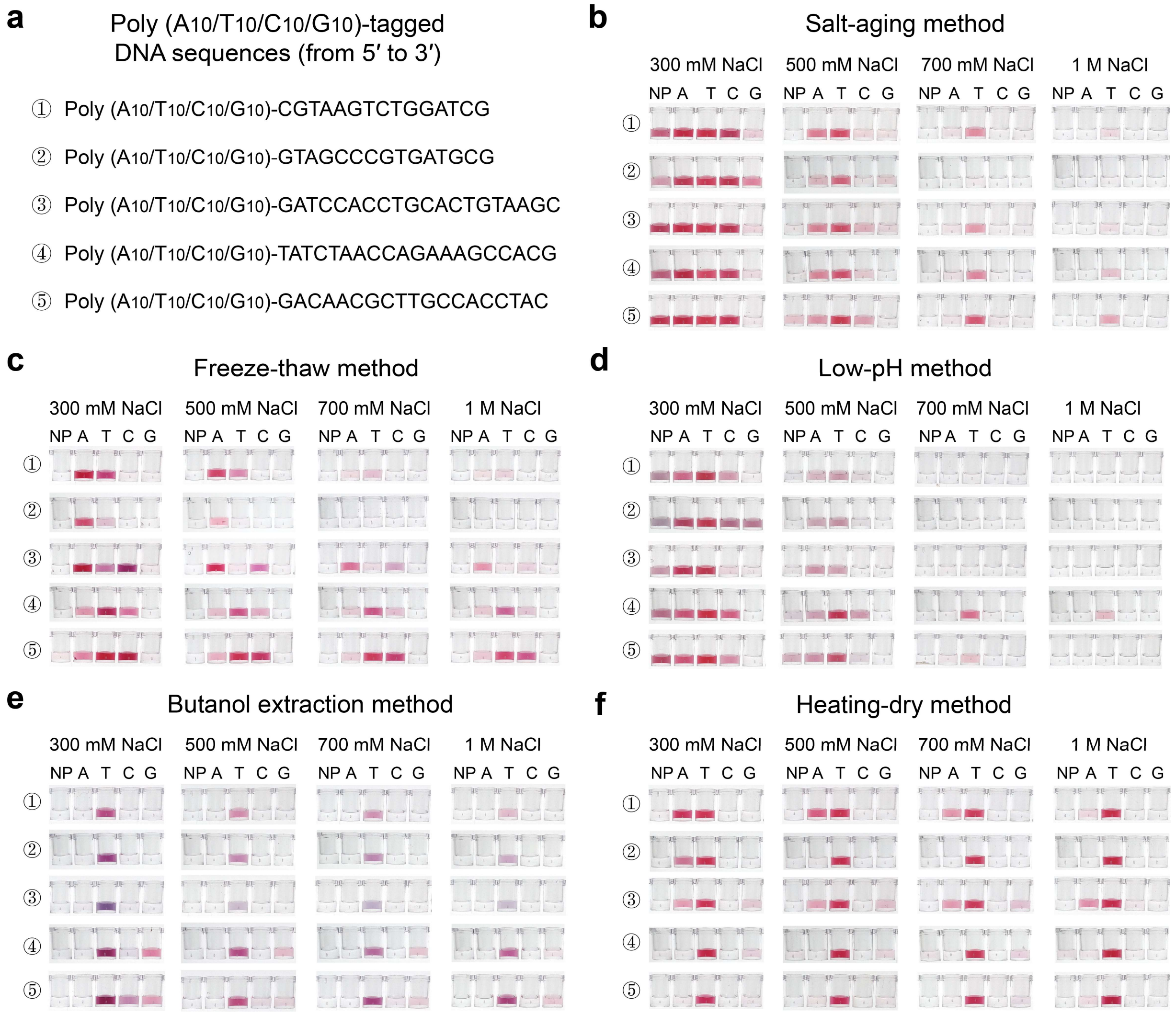

Supplementary Fig. 21 Non-thiolated DNA-AuNP conjugation with various labeling methods. **(a)** Detailed sequences of six poly ($A_{10}/T_{10}/C_{10}/G_{10}$)-tagged DNA probes. Photographs showing the labeling results of the six poly ($A_{10}/T_{10}/C_{10}/G_{10}$)-tagged DNA probes using **(b)** salt-aging method. **(c)** freeze-thaw method, **(d)** low-pH method, **(e)** butanol extraction method, and **(f)** MW-assisted heating-dry method. The DNA-AuNP conjugates were resuspended in 300 mM, 500 mM, 700 mM and 1 M NaCl solution, respectively. MW-assisted heating-dry method exhibits maximum labeling efficiency and excellent sequence universality compared with other methods.

Supplementary Table 1 The mean hydrodynamic diameter and polydispersity index (PDI).

Names	Average hydrodynamic diameter (nm)	Polydispersity index (PDI)
**AuNPs**	22.7	0.18
**Poly (T)-tagged DNA-AuNP**	36.4	0.19
**Poly (U)-tagged sgRNA-AuNP**	161.7	0.32
**Poly (U)-tagged *N* gene-RNA-AuNP**	285.4	0.22

Supplementary Table 2 DNA and RNA sequences used in this work.[a]

Name	Sequences (from 5′ to 3′)	Locations
DNA	CGTAAGTCTGGATCG	Fig. 1b, Supplementary Fig. 1a
Poly (T_{10})-DNA (5′)	TTTTTTTTTTCGTAAGTCTGGATCG	Fig. 1b-e, j, Fig. 2a, b, Fig. 5a, Supplementary Figs. 15, 16a
FAM-poly (T_{10})-DNA	TTTTTTTTTTCCCCC-FAM	Fig. 1f-i, Supplementary Figs. 2a-e, 5a, 12
FAM-poly (A_{10})-DNA	AAAAAAAAAATTTTT-FAM	Fig. 1f, Supplementary Fig. 5b
Poly (rU_{10})-RNA	UUUUUUUUUUCGUAAGUCUGGAUCG	Fig. 1j, Fig. 2a
Poly(A_{10})-DNA (5′)	AAAAAAAAAACGTAAGTCTGGATCG	Fig. 2a, Fig. 5a, Supplementary Figs. 7a, 15, 21
Poly (C_{10})-DNA (5′)	CCCCCCCCCCCGTAAGTCTGGATCG	Fig. 2a, Fig. 5a, Supplementary Figs. 9b, 15
Poly (G_{10})-DNA (5′)	GGGGGGGGGGCGTAAGTCTGGATCG	Fig. 2a, Fig. 5a, Supplementary Figs. 9c, 15
Poly (A_{10})-DNA (3′)	GCTAGGTCTGAATGCAAAAAAAAAA	Fig. 2a Supplementary Fig. 9a
Poly (T_{10})-DNA (3′)	GCTAGGTCTGAATGCTTTTTTTTTT	Fig. 2a, b
Poly (C_{10})-DNA (3′)	GCTAGGTCTGAATGCCCCCCCCCCC	Fig. 2a, Supplementary Fig. 9b
Poly (G_{10})-DNA (3′)	GCTAGGTCTGAATGCGGGGGGGGGG	Fig. 2a, Supplementary Fig. 9c
Poly (T_{10})-DNA-M	CGCGTTGAATCTATCTTTTTTTTTTCGTAAGT CTGGATCG	Fig. 2a, b
Poly (A_{10})-DNA-M	CGCGTTGAATCTATCAAAAAAAAAACGTAA GTCTGGATCG	Fig. 2a, Supplementary Fig. 9a
Poly (C_{10})-DNA-M	CGCGTTGAATCTATCCCCCCCCCCCCGTAAG TCTGGATCG	Fig. 2a, Supplementary Fig. 9b
Poly (G_{10})-DNA-M	CGCGTTGAATCTATCGGGGGGGGGGCGTAA GTCTGGATCG	Fig. 2a, Supplementary Fig. 9c
RNA	CGUAAGUCUGGAUCG	Fig. 2a
Poly (rA_{10})-RNA	AAAAAAAAAACGUAAGUCUGGAUCG	Fig. 2a
Poly (rC_{10})-RNA	CCCCCCCCCCCGUAAGUCUGGAUCG	Fig. 2a
Poly (rG_{10})-RNA	GGGGGGGGGGCGUAAGUCUGGAUCG	Fig. 2a
Poly (T_5)-DNA (5′)	TTTTTCGTAAGTCTGGATCG	Fig. 2b, c
Poly (T_{15})-DNA (5′)	TTTTTTTTTTTTTTTCGTAAGTCTGGATCG	Fig. 2b
Poly (T_{20})-DNA (5′)	TTTTTTTTTTTTTTTTTTTTCGTAAGTCTGGA TCG	Fig. 2b
Poly (T_{25})-DNA (5′)	TTTTTTTTTTTTTTTTTTTTTTTTTCGTAAGT CTGGATCG	Fig. 2b

Poly (T_{30})-DNA (5′)	TTTTTTTTTTTTTTTTTTTTTTTTTTTTTTCGT AAGTCTGGATCG	Fig. 2b
Poly (T_5)-DNA (3′)	GCTAGGTCTGAATGCTTTTT	Fig. 2b, c
Poly (T_{15})-DNA (3′)	GCTAGGTCTGAATGCTTTTTTTTTTTTTTT	Fig. 2b
Poly (T_{20})-DNA (3′)	GCTAGGTCTGAATGCTTTTTTTTTTTTTTTTT TTT	Fig. 2b
Poly (T_{25})-DNA (3′)	GCTAGGTCTGAATGCTTTTTTTTTTTTTTTTT TTTTTTTT	Fig. 2b
Poly (T_{30})-DNA (3′)	GCTAGGTCTGAATGCTTTTTTTTTTTTTTTTT TTTTTTTTTTTTT	Fig. 2b
Poly (T_5)-DNA-M	CGCGTTGAATCTATCTTTTTCGTAAGTCTGG ATCG	Fig. 2b
Poly (T_{15})-DNA-M	CGCGTTGAATCTATCTTTTTTTTTTTTTTTCG TAAGTCTGGATCG	Fig. 2b
Poly (T_{20})-DNA-M	CGCGTTGAATCTATCTTTTTTTTTTTTTTTTT TTTCGTAAGTCTGGATCG	Fig. 2b
Poly (T_{25})-DNA-M	CGCGTTGAATCTATCTTTTTTTTTTTTTTTTT TTTTTTTTCGTAAGTCTGGATCG	Fig. 2b
Poly (T_{30})-DNA-M	CGCGTTGAATCTATCTTTTTTTTTTTTTTTTT TTTTTTTTTTTTTCGTAAGTCTGGATCG	Fig. 2b
T_3C_2-1-DNA (5′)	CCTTTCGTAAGTCTGGATCG	Fig. 2c
T_3C_2-2-DNA (5′)	TCCTTCGTAAGTCTGGATCG	Fig. 2c
T_3C_2-3-DNA (5′)	TTTCCCGTAAGTCTGGATCG	Fig. 2c
T_4C_1-1-DNA (5′)	CTTTTCGTAAGTCTGGATCG	Fig. 2c
T_4C_1-2-DNA (5′)	TCTTTCGTAAGTCTGGATCG	Fig. 2c
T_4C_1-3-DNA (5′)	TTCTTCGTAAGTCTGGATCG	Fig. 2c
T_4C_1-4-DNA (5′)	TTTCTCGTAAGTCTGGATCG	Fig. 2c
T_4C_1-5-DNA (5′)	TTTTCCGTAAGTCTGGATCG	Fig. 2c
T_3C_2-1-DNA (3′)	GCTAGGTCTGAATGCTTTCC	Fig. 2c
T_3C_2-2-DNA (3′)	GCTAGGTCTGAATGCTTCCT	Fig. 2c
T_3C_2-3-DNA (3′)	GCTAGGTCTGAATGCCCTTT	Fig. 2c
T_4C_1-1-DNA (3′)	GCTAGGTCTGAATGCTTTTC	Fig. 2c
T_4C_1-2-DNA (3′)	GCTAGGTCTGAATGCTTTCT	Fig. 2c
T_4C_1-3-DNA (3′)	GCTAGGTCTGAATGCTTCTT	Fig. 2c
T_4C_1-4-DNA (3′)	GCTAGGTCTGAATGCTCTTT	Fig. 2c
T_4C_1-5-DNA (3′)	GCTAGGTCTGAATGCCTTTT	Fig. 2c
T_3A_2-1-DNA (5′)	AATTTCGTAAGTCTGGATCG	Fig. 2c
T_3A_2-2-DNA (5′)	TAATTCGTAAGTCTGGATCG	Fig. 2c
T_3A_2-3-DNA (5′)	TTTAACGTAAGTCTGGATCG	Fig. 2c
T_4A_1-1-DNA (5′)	ATTTTCGTAAGTCTGGATCG	Fig. 2c
T_4A_1-2-DNA (5′)	TATTTCGTAAGTCTGGATCG	Fig. 2c
T_4A_1-3-DNA (5′)	TTATTCGTAAGTCTGGATCG	Fig. 2c
T_4A_1-4-DNA (5′)	TTTATCGTAAGTCTGGATCG	Fig. 2c
T_4A_1-5-DNA (5′)	TTTTACGTAAGTCTGGATCG	Fig. 2c
T_3A_2-1-DNA (3′)	GCTAGGTCTGAATGCTTTAA	Fig. 2c
T_3A_2-2-DNA (3′)	GCTAGGTCTGAATGCTTAAT	Fig. 2c
T_3A_2-3-DNA (3′)	GCTAGGTCTGAATGCAATTT	Fig. 2c
T_4A_1-1-DNA (3′)	GCTAGGTCTGAATGCTTTTA	Fig. 2c
T_4A_1-2-DNA (3′)	GCTAGGTCTGAATGCTTTAT	Fig. 2c
T_4A_1-3-DNA (3′)	GCTAGGTCTGAATGCTTATT	Fig. 2c

T_4A_1-4-DNA (3′)	GCTAGGTCTGAATGCTATTT	Fig. 2c
T_4A_1-5-DNA (3′)	GCTAGGTCTGAATGCATTTT	Fig. 2c
T_3G_2-1-DNA (5′)	GGTTTCGTAAGTCTGGATCG	Fig. 2c
T_3G_2-2-DNA (5′)	TGGTTCGTAAGTCTGGATCG	Fig. 2c
T_3G_2-3-DNA (5′)	TTTGGCGTAAGTCTGGATCG	Fig. 2c
T_4G_1-1-DNA (5′)	GTTTTCGTAAGTCTGGATCG	Fig. 2c
T_4G_1-2-DNA (5′)	TGTTTCGTAAGTCTGGATCG	Fig. 2c
T_4G_1-3-DNA (5′)	TTGTTCGTAAGTCTGGATCG	Fig. 2c
T_4G_1-4-DNA (5′)	TTTGTCGTAAGTCTGGATCG	Fig. 2c
T_4G_1-5-DNA (5′)	TTTTGCGTAAGTCTGGATCG	Fig. 2c
T_3G_2-1-DNA (3′)	GCTAGGTCTGAATGCTTTGG	Fig. 2c
T_3G_2-2-DNA (3′)	GCTAGGTCTGAATGCTTGGT	Fig. 2c
T_3G_2-3-DNA (3′)	GCTAGGTCTGAATGCGGTTT	Fig. 2c
T_4G_1-1-DNA (3′)	GCTAGGTCTGAATGCTTTTG	Fig. 2c
T_4G_1-2-DNA (3′)	GCTAGGTCTGAATGCTTTGT	Fig. 2c
T_4G_1-3-DNA (3′)	GCTAGGTCTGAATGCTTGTT	Fig. 2c
T_4G_1-4-DNA (3′)	GCTAGGTCTGAATGCTGTTT	Fig. 2c
T_4C_1-5-DNA (3′)	GCTAGGTCTGAATGCGTTTT	Fig. 2c
$T_{10}A_1$	TTTTTTTTTTA	Fig. 2d
$T_{10}T_1$	TTTTTTTTTTT	Fig. 2d
$T_{10}C_1$	TTTTTTTTTTC	Fig. 2d
$T_{10}G_1$	TTTTTTTTTTG	Fig. 2d
$T_{10}A_2$	TTTTTTTTTTAA	Fig. 2d
$T_{10}T_2$	TTTTTTTTTTTT	Fig. 2d
$T_{10}C_2$	TTTTTTTTTTCC	Fig. 2d
$T_{10}G_2$	TTTTTTTTTTGG	Fig. 2d
$A_{10}A_2$	AAAAAAAAAAAA	Fig. 2d
$A_{10}T_2$	AAAAAAAAAATT	Fig. 2d
$A_{10}C_2$	AAAAAAAAAACC	Fig. 2d
$A_{10}G_2$	AAAAAAAAAAGG	Fig. 2d
$C_{10}A_2$	CCCCCCCCCCAA	Fig. 2d
$C_{10}T_2$	CCCCCCCCCCTT	Fig. 2d
$C_{10}C_2$	CCCCCCCCCCCC	Fig. 2d
$C_{10}G_2$	CCCCCCCCCCGG	Fig. 2d
$G_{10}A_2$	GGGGGGGGGGAA	Fig. 2d
$G_{10}T_2$	GGGGGGGGGGTT	Fig. 2d
$G_{10}C_2$	GGGGGGGGGGCC	Fig. 2d
$G_{10}G_2$	GGGGGGGGGGGG	Fig. 2d
SH-DNA	SH-GCTTACAGTGCAGGTGGATCCGATAC AGACTTACG	Fig. 3a
A_5-DNA-T_5	AAAAAGCTTACAGTGCAGGTGGATCCGATA CAGACTTACGTTTTT	Fig. 3a

A_5-DNA-T_{10}	AAAAAGCTTACAGTGCAGGTGGATCCGATACAGACTTACGTTTTTTTTTT	Fig. 3a
T_5-DNA-A_5	TTTTTGCTTACAGTGCAGGTGGATCCGATACAGACTTACGAAAAA	Fig. 3a
T_{10}-DNA-A_5	TTTTTTTTTTGCTTACAGTGCAGGTGGATCCGATACAGACTTACGAAAAA	Fig. 3a
ROX-cDNA	ROX-CGTAAGTCTGTATCGGATCCACCTGCACTGTAAGC	Fig. 3a
T_{10}-G4-DNA1	TTTTTTTTTTGGTGGTGGTGGTGGTGGTGGT	Fig. 3b
T_{10}-G4-DNA1-T_{10}	TTTTTTTTTTGGTGGTGGTGGTGGTGGTGGTTTTTTTTTT	Fig. 3b
T_{10}-G4-DNA1-A_5	TTTTTTTTTTGGTGGTGGTGGTGGTGGTGGTTTAAAAA	Fig. 3b
T_{10}-G4-DNA1-A_{10}	TTTTTTTTTTGGTGGTGGTGGTGGTGGTGGTTTAAAAAAAAAA	Fig. 3b
T_{10}-G4-DNA1-A_{15}	TTTTTTTTTTGGTGGTGGTGGTGGTGGTGGTTTAAAAAAAAAAAAAAA	Fig. 3b
T_{10}-G4-DNA2	TTTTTTTTTTGGGTAGGGCGGGTTGGG	Fig. 3c
T_{10}-G4-DNA2-T_{10}	TTTTTTTTTTGGGTAGGGCGGGTTGGGTTTTTTTTTT	Fig. 3c
T_{10}-G4-DNA2-A_5	TTTTTTTTTTGGGTAGGGCGGGTTGGGTTTAAAAA	Fig. 3c
T_{10}-G4-DNA2-A_{10}	TTTTTTTTTTGGGTAGGGCGGGTTGGGTTTAAAAAAAAAA	Fig. 3c
T_{10}-G4-DNA2-A_{15}	TTTTTTTTTTGGGTAGGGCGGGTTGGGTTTAAAAAAAAAAAAAAA	Fig. 3c
Poly (T_5)-DNA (hybrid)	TTTTTCATTGCTAGAGCAGG	Fig. 4a
Poly (T_{10})-DNA (hybrid)	TTTTTTTTTTCATTGCTAGAGCAGG	Fig. 4a
Poly (T_{15})-DNA (hybrid)	TTTTTTTTTTTTTTTCATTGCTAGAGCAGG	Fig. 4a
Poly (T_{20})-DNA (hybrid)	TTTTTTTTTTTTTTTTTTTTCATTGCTAGAGCAGG	Fig. 4a
Poly (T_{25})-DNA (hybrid)	TTTTTTTTTTTTTTTTTTTTTTTTTCATTGCTAGAGCAGG	Fig. 4a
Poly (T_{30})-DNA (hybrid)	TTTTTTTTTTTTTTTTTTTTTTTTTTTTTTCATTGCTAGAGCAGG	Fig. 4a
Poly (T_7)-DNA-poly(A_0) (hybrid)	TTTTTTTCATTGCTAGAGCAGG	Fig. 4a
Poly (T_7)-DNA-poly(A_2) (hybrid)	TTTTTTTCATTGCTAGAGCAGGAA	Fig. 4a
Poly (T_7)-DNA-poly(A_5) (hybrid)	TTTTTTTCATTGCTAGAGCAGGAAAAA	Fig. 4a
Poly (T_7)-DNA-poly(A_{10}) (hybrid)	TTTTTTTCATTGCTAGAGCAGGAAAAAAAAAA	Fig. 4a
Poly (T_7)-DNA-poly(A_{15}) (hybrid)	TTTTTTTCATTGCTAGAGCAGGAAAAAAAAAAAAAAA	Fig. 4a
Poly (T_7)-DNA-poly(A_{20}) (hybrid)	TTTTTTTCATTGCTAGAGCAGGAAAAAAAAAAAAAAAAAAAA	Fig. 4a
Poly (T_7)-DNA-poly(A_{25}) (hybrid)	TTTTTTTCATTGCTAGAGCAGGAAAAAAAAAAAAAAAAAAAAAAAAA	Fig. 4a

Poly (T_5)-Poly(C_5)-FAM	TTTTTCCCCC-FAM	Fig. 4b, Supplementary Fig. 12
Poly (T_6)-Poly(C_5)-FAM	TTTTTTCCCCC-FAM	Fig. 4b, Supplementary Fig. 12
Poly (T_7)-Poly(C_5)-FAM	TTTTTTTCCCCC-FAM	Fig. 4b, Supplementary Fig. 12
Poly (T_8)-Poly(C_5)-FAM	TTTTTTTTCCCCC-FAM	Fig. 4b, Supplementary Fig. 12
Poly (T_9)-Poly(C_5)-FAM	TTTTTTTTTCCCCC-FAM	Fig. 4b, Supplementary Fig. 12
Poly (T_{15})-Poly (C_5)-FAM	TTTTTTTTTTTTTTTCCCCC-FAM	Fig. 4b, Supplementary Fig. 12
Poly (T_{20})-Poly (C_5)-FAM	TTTTTTTTTTTTTTTTTTTTCCCCC-FAM	Fig. 4b, Supplementary Fig. 12
Poly (T_{25})-Poly (C_5)-FAM	TTTTTTTTTTTTTTTTTTTTTTTTTCCCCC-FAM	Fig. 4b, Supplementary Fig. 10
Poly (T_{30})-Poly (C_5)-FAM	TTTTTTTTTTTTTTTTTTTTTTTTTTTTTTCCCCC-FAM	Fig. 4b, Supplementary Fig. 12
Poly (T_{10})-Poly (C_2)-FAM	TTTTTTTTTTCC-FAM	Fig. 4b, Supplementary Fig. 13
Poly (T_{10})-Poly (C_7)-FAM	TTTTTTTTTTCCCCCCC-FAM	Fig. 4b, Supplementary Fig. 13
Poly (T_{10})-Poly (C_{10})-FAM	TTTTTTTTTTCCCCCCCCCC-FAM	Fig. 4b, Supplementary Fig. 13
Poly (T_{10})-Poly (C_{15})-FAM	TTTTTTTTTTCCCCCCCCCCCCCCC-FAM	Fig. 4b, Supplementary Fig. 13
Poly (T_{10})-Poly (C_{20})-FAM	TTTTTTTTTTCCCCCCCCCCCCCCCCCCCC-FAM	Fig. 4b, Supplementary Fig. 13
Poly (T_{10})-Poly (C_{25})-FAM	TTTTTTTTTTCCCCCCCCCCCCCCCCCCCCCCCCC-FAM	Fig. 4b, Supplementary Fig. 14
Poly (T_{10})-Poly (A_2)-FAM	TTTTTTTTTTAA-FAM	Fig. 4b, Supplementary Fig. 14
Poly (T_{10})-Poly (A_7)-FAM	TTTTTTTTTTAAAAAAA-FAM	Fig. 4b, Supplementary Fig. 14
Poly (T_{10})-Poly (A_{10})-FAM	TTTTTTTTTTAAAAAAAAAA-FAM	Fig. 4b, Supplementary Fig. 14
Poly (T_{10})-Poly (A_{15})-FAM	TTTTTTTTTTAAAAAAAAAAAAAAA-FAM	Fig. 4b, Supplementary Fig. 14
Poly (T_{10})-Poly (A_{20})-FAM	TTTTTTTTTTAAAAAAAAAAAAAAAAAAAA-FAM	Fig. 4b, Supplementary Fig. 14
Poly (T_{10})-Poly (A_{25})-FAM	TTTTTTTTTTAAAAAAAAAAAAAAAAAAAAAAAAA-FAM	Fig. 4b, Supplementary Fig. 14
DNA (1)	TATCTAACCAGAAAGCCACG	Fig. 5a, Supplementary Figs. 15, 21
Poly (A_{10})-DNA (1)	AAAAAAAAAATATCTAACCAGAAAGCCACG	Fig. 5a, Supplementary Figs. 15, 21
Poly (T_{10})-DNA (1)	TTTTTTTTTTTATCTAACCAGAAAGCCACG	Fig. 5a, Supplementary Figs. 15, 21

Poly (C_{10})-DNA (1)	CCCCCCCCCCTATCTAACCAGAAAGCCACG	Fig. 5a, Supplementary Figs. 15, 21
Poly (G_{10})-DNA (1)	GGGGGGGGGGTATCTAACCAGAAAGCCACG	Fig. 5a, Supplementary Figs. 15, 21
DNA (2)	GATCCACCTGCACTGTAAGC	Fig. 5a, Supplementary Figs. 15, 21
Poly (A_{10})-DNA (2)	AAAAAAAAAAGATCCACCTGCACTGTAAGC	Fig. 5a, Supplementary Figs. 15, 21
Poly (T_{10})-DNA (2)	TTTTTTTTTTGATCCACCTGCACTGTAAGC	Fig. 5a, Supplementary Figs. 15, 21
Poly (C_{10})-DNA (2)	CCCCCCCCCCGATCCACCTGCACTGTAAGC	Fig. 5a, Supplementary Figs. 15, 21
Poly (G_{10})-DNA (2)	GGGGGGGGGGGATCCACCTGCACTGTAAGC	Fig. 5a, Supplementary Figs. 15, 21
DNA (3)	GTAGCCCGTG ATGCG	Fig. 5a, Supplementary Figs. 15, 21
Poly (A_{10})-DNA (3)	AAAAAAAAAAGTAGCCCGTG ATGCG	Fig. 5a, Supplementary Figs. 15, 21
Poly (T_{10})-DNA (3)	TTTTTTTTTTGTAGCCCGTG ATGCG	Fig. 5a, Supplementary Figs. 15, 21
Poly (C_{10})-DNA (3)	CCCCCCCCCCGTAGCCCGTG ATGCG	Fig. 5a, Supplementary Figs. 15, 21
Poly (G_{10})-DNA (3)	GGGGGGGGGGGTAGCCCGTG ATGCG	Fig. 5a, Supplementary Figs. 15, 21
Padlock probe (with A_{40})	AAGAACTATATTGAAAAAAAAAAAAAAAAAAAAAAAAAAAAAAAAAAAAAAAAAAACACCTTCTCA	Fig. 6a-f
Padlock probe (random)	AAGAACTATATTGAGCTTACAGTGCAGCTGGATCGTAGTGGCAAGCGTTGTCACACCTTCTCA	Fig. 6a-c
CFTR *G542X* Locus (Mutant type)	GACAATATAGTTCTTTGAGAAGGTG	Fig. 6a-f
CFTR *G542X* Locus (Wild type)	GACAATATAGTTCTTGGAGAAGGTG	Fig. 6d-f
sgDNA primer-F	GAAATTAATACGACTCACTATAGGGAGATACGTTGCGTCCGTGATGTTTTAGAGCTAGAAATAGCAAG	Fig. 6g-o, Supplementary Fig. 19a, b
sgDNA primer-R-poly (A_{17})	AAAAAAAAAAAAAAAAAGTAGGTGGCAAGCGTTGTCAGCACCGACTCGGTGCCACTTT	Fig. 6g-o, Supplementary Fig. 19a, b

	TTCAAGTTGATAACGGACTAGCCTTATTTTA ACTTGCTATTTCTAGC	
sgDNA primer-R	GTAGGTGGCAAGCGTTGTCAGCACCGACTC GGTGCCACTTTTTCAAGTTGATAACGGACT AGCCTTATTTTAACTTGCTATTTCTAGC	Fig. 6j
N gene-primer-F	GAAATTAATACGACTCACTATAGGGATGTCT GATAATGGACCCCA	Fig. 6g-l
N gene-primer-R-poly (A_{15})	AAAAAAAAAAAAAAATTAGGCCTGAGTTG AGTCA	Fig. 6g-l
N gene-primer-R	TTAGGCCTGAGTTGAGTCA	Fig. 6j
Capture DNA (ASFV)	ATCACGGACGCAACGTATCTTTTT-Bio	Fig. 6n, o
ASFV-primer-F	Bio-ATGGATACTGAGGGAATAGC	Fig. 6 n, o, Supplementary Fig. 19a, b
ASFV-primer-R	CTTACCGATGAAAATGATAC	Fig. 6n, o, Supplementary Fig. 19a, b
SH-DNA	SH-CGTAAGTCTGGATCG	Supplementary Fig. 1a, 6a-e, 16b
SH-DNA-FAM	SH-TTTTTTTTTT-FAM	Supplementary Fig. 5c, d
Capture DNA	Bio-CCTGCTCTAGCAATG	Supplementary Fig. 6a, b
Poly (T_5)-DNA (hybrid)	TTTTTCATTGCTAGAGCAGG	Supplementary Fig. 6a
SH-DNA (hybrid)	SH-CATTGCTAGAGCAGG	Supplementary Fig. 6b
Primer-F	CTAGTATGGCTGGCAATGGCG	Supplementary Fig. 7
Primer-R	GCTCTCAAGCTGGTTCAATCTGTC	Supplementary Fig. 7
DNA template	CTAGTATGGCTGGCAATGGCGGTGATGTAC GTTCTCTATGACTACTGCATCAGACTTCAGT CCAGAGCTGCAGTGCTCATCTGAGCTGACA GTCAGGACAGATTGAACCAGCTTGAGAGC	Supplementary Fig. 7
ssDNA	AGTCACTGCCATGCATTCGTCGAGACCTAG CTTAC	Supplementary Fig. 8
C-ssDNA	GTAAGCTAGGTCTCGACGAATGCATGGCAG TGACT	Supplementary Fig. 8
Poly (A_5)-DNA (5′)	AAAAACGTAAGTCTGGATCG	Supplementary Fig. 9a
Poly (A_{15})-DNA (5′)	AAAAAAAAAAAAAAACGTAAGTCTGGATC G	Supplementary Fig. 9a
Poly (A_{20})-DNA (5′)	AAAAAAAAAAAAAAAAAAAACGTAAGTCT GGATCG	Supplementary Fig. 9a
Poly (A_{25})-DNA (5′)	AAAAAAAAAAAAAAAAAAAAAAAAACGT AAGTCTGGATCG	Supplementary Fig. 9a
Poly (A_{30})-DNA (5′)	AAAAAAAAAAAAAAAAAAAAAAAAAAAA AACGTAAGTCTGGATCG	Supplementary Fig. 9a
Poly (A_5)-DNA (3′)	GCTAGGTCTGAATGCAAAAA	Supplementary Fig. 9a
Poly (A_{15})-DNA (3′)	GCTAGGTCTGAATGCAAAAAAAAAAAAAA A	Supplementary Fig. 9a
Poly (A_{20})-DNA (3′)	GCTAGGTCTGAATGCAAAAAAAAAAAAAA AAAAAA	Supplementary Fig. 9a
Poly (A_{25})-DNA (3′)	GCTAGGTCTGAATGCAAAAAAAAAAAAAA AAAAAAAAAAA	Supplementary Fig. 9a

Poly (A_{30})-DNA (3′)	GCTAGGTCTGAATGCAAAAAAAAAAAAAA<br>AAAAAAAAAAAAAAAA	Supplementary Fig. 9a
Poly (A_5)-DNA-M	CGCGTTGAATCTATCAAAAACGTAAGTCTG<br>GATCG	Supplementary Fig. 9a
Poly (A_{15})-DNA-M	CGCGTTGAATCTATCAAAAAAAAAAAAAA<br>ACGTAAGTCTGGATCG	Supplementary Fig. 9a
Poly (A_{20})-DNA-M	CGCGTTGAATCTATCAAAAAAAAAAAAAA<br>AAAAAACGTAAGTCTGGATCG	Supplementary Fig. 9a
Poly (A_{25})-DNA-M	CGCGTTGAATCTATCAAAAAAAAAAAAAA<br>AAAAAAAAAAACGTAAGTCTGGATCG	Supplementary Fig. 9a
Poly (A_{30})-DNA-M	CGCGTTGAATCTATCAAAAAAAAAAAAAA<br>AAAAAAAAAAAAAAAACGTAAGTCTGGAT<br>CG	Supplementary Fig. 9a
Poly (C_5)-DNA (5′)	CCCCCCGTAAGTCTGGATCG	Supplementary Fig. 9b
Poly (C_{15})-DN (5′)	CCCCCCCCCCCCCCCCGTAAGTCTGGATCG	Supplementary Fig. 9b
Poly (C_{20})-DNA (5′)	CCCCCCCCCCCCCCCCCCCCCGTAAGTCTG<br>GATCG	Supplementary Fig. 9b
Poly (C_{25})-DNA (5′)	CCCCCCCCCCCCCCCCCCCCCCCCCCGTAA<br>GTCTGGATCG	Supplementary Fig. 9b
Poly (C_{30})-DNA (5′)	CCCCCCCCCCCCCCCCCCCCCCCCCCCCCC<br>CGTAAGTCTGGATCG	Supplementary Fig. 9b
Poly (C_5)-DNA (3′)	GCTAGGTCTGAATGCCCCCC	Supplementary Fig. 9b
Poly (C_{15})-DNA (3′)	GCTAGGTCTGAATGCCCCCCCCCCCCCCCC	Supplementary Fig. 9b
Poly (C_{20})-DNA (3′)	GCTAGGTCTGAATGCCCCCCCCCCCCCCCC<br>CCCCC	Supplementary Fig. 9b
Poly (C_{25})-DNA (3′)	GCTAGGTCTGAATGCCCCCCCCCCCCCCCC<br>CCCCCCCCCC	Supplementary Fig. 9b
Poly (C_{30})-DNA (3′)	GCTAGGTCTGAATGCCCCCCCCCCCCCCCC<br>CCCCCCCCCCCCCCC	Supplementary Fig. 9b
Poly (C_5)-DNA-M	CGCGTTGAATCTATCCCCCCGTAAGTCTGG<br>ATCG	Supplementary Fig. 9b
Poly (C_{15})-DNA-M	CGCGTTGAATCTATCCCCCCCCCCCCCCCC<br>GTAAGTCTGGATCG	Supplementary Fig. 9b
Poly (C_{20})-DNA-M	CGCGTTGAATCTATCCCCCCCCCCCCCCCC<br>CCCCCGTAAGTCTGGATCG	Supplementary Fig. 9b
Poly (C_{25})-DNA-M	CGCGTTGAATCTATCCCCCCCCCCCCCCCC<br>CCCCCCCCCCGTAAGTCTGGATCG	Supplementary Fig. 9b
Poly (C_{30})-DNA-M	CGCGTTGAATCTATCCCCCCCCCCCCCCCC<br>CCCCCCCCCCCCCCCGTAAGTCTGGATCG	Supplementary Fig. 9b
Poly (G_5)-DNA (5′)	GGGGGCGTAAGTCTGGATCG	Supplementary Fig. 9c
Poly (G_5)-DNA (3′)	GCTAGGTCTGAATGCGGGGG	Supplementary Fig. 9c
Poly (G_5)-DNA-M	CGCGTTGAATCTATCGGGGGCGTAAGTCTG<br>GATCG	Supplementary Fig. 9c
T_{10}-DNA-T_{10}	TTTTTTTTTTCGTAAGTCTGGATCGTTTTTTT<br>TTT	Supplementary Fig.<br>10a
Hairpin DNA1	TTTTTTTCGCGTTGAATCTATCAAAAAAA	Supplementary Fig.<br>10b, c
Hairpin DNA2	TTTTTTTCGTAAGTCTGGATCGAAAAAAA	Supplementary Fig.<br>10c
Hairpin DNA3	TTTTTTTGTAGCCCGTGATGCGAAAAAAA	Supplementary Fig.<br>10c

Hairpin DNA4	TTTTTTTGATCCACCTGCACTGTAAGCAAAAAAA	Supplementary Fig. 10c
Hairpin DNA5	TTTTTTTTATCTAACCAGAAAGCCACGAAAAAAA	Supplementary Fig. 10c
Hairpin DNA6	TTTTTTTGACAACGCTTGCCACCTACAAAAAAA	Supplementary Fig. 10c
DNA (4)	GATCCACCTGCACTGTAAGC	Supplementary Figs. 15, 21
Poly (A_{10})-DNA (4)	AAAAAAAAAAGATCCACCTGCACTGTAAGC	Supplementary Figs. 15, 21
Poly (T_{10})-DNA (4)	TTTTTTTTTTGATCCACCTGCACTGTAAGC	Supplementary Figs. 15, 21
Poly (C_{10})-DNA (4)	CCCCCCCCCCGATCCACCTGCACTGTAAGC	Supplementary Figs. 15, 21
Poly (G_{10})-DNA (4)	GGGGGGGGGGGATCCACCTGCACTGTAAGC	Supplementary Figs. 15, 21
DNA (5)	CGCGTTGAATCTATC	Supplementary Figs. 15, 21
Poly (A_{10})-DNA (5)	AAAAAAAAAACGCGTTGAATCTATC	Supplementary Figs. 15, 21
Poly (T_{10})-DNA (5)	TTTTTTTTTTCGCGTTGAATCTATCA	Supplementary Figs. 15, 21
Poly (C_{10})-DNA (5)	CCCCCCCCCCCGCGTTGAATCTATC	Supplementary Figs. 15, 21
Poly (G_{10})-DNA (5)	GGGGGGGGGGCGCGTTGAATCTATC	Supplementary Figs. 15, 21
DNA (6)	GACAACGCTTGCCACCTAC	Supplementary Figs. 15, 21
Poly (A_{10})-DNA (6)	AAAAAAAAAAGACAACGCTTGCCACCTAC	Supplementary Figs. 15, 21
Poly (T_{10})-DNA (6)	TTTTTTTTTTGACAACGCTTGCCACCTAC	Supplementary Figs. 15, 21
Poly (C_{10})-DNA (6)	CCCCCCCCCCGACAACGCTTGCCACCTAC	Supplementary Figs. 15, 21
Poly (G_{10})-DNA (6)	GGGGGGGGGGGACAACGCTTGCCACCTAC	Supplementary Figs. 15, 21
Poly (T^*_5)-DNA	T*T*T*T*T*CATTGCTAGAGCAGG	Supplementary Fig. 17b
Poly (T^*_7)-DNA	T*T*T*T*T*T*T*CATTGCTAGAGCAGG	Supplementary Fig. 17b
Poly (T^*_{10})-DNA	T*T*T*T*T*T*T*T*T*T*CATTGCTAGAGCAGG	Supplementary Fig. 17b
Poly (T^*_{15})-DNA	T*T*T*T*T*T*T*T*T*T*T*T*T*T*T*CATTGCTAGAGCAGG	Supplementary Fig. 17b
Poly (T^*_7)-DNA-Poly (T_7)	T*T*T*T*T*T*T*CATTGCTAGAGCAGGTTTTTTT	Supplementary Fig. 17b

[a]M stands for the middle region. The mutant base is highlighted in red. * stands for the phosphorothioate (PS) modification. CTFR stands for human cystic fibrosis transmembrane conductance regulator.

Supplementary Table 3 Genomic sequences used in this work.

Names	Sequences (from 5′ to 3′)
N gene (*GU280_gp10*) of SARS-CoV-2 (accession: NC_045512.2)	ATGTCTGATAATGGACCCCAAAATCAGCGAAATGCACCCCGCA<br>TTACGTTTGGTGGACCCTCAGATTCAACTGGCAGTAACCAGAA<br>TGGAGAACGCAGTGGGGCGCGATCAAAACAACGTCGGCCCCA<br>AGGTTTACCCAATAATACTGCGTCTTGGTTCACCGCTCTCACTC<br>AACATGGCAAGGAAGACCTTAAATTCCCTCGAGGACAAGGCG<br>TTCCAATTAACACCAATAGCAGTCCAGATGACCAAATTGGCTAC<br>TACCGAAGAGCTACCAGACGAATTCGTGGTGGTGACGGTAAA<br>ATGAAAGATCTCAGTCCAAGATGGTATTTCTACTACCTAGGAAC<br>TGGGCCAGAAGCTGGACTTCCCTATGGTGCTAACAAAGACGGC<br>ATCATATGGGTTGCAACTGAGGGAGCCTTGAATACACCAAAAG<br>ATCACATTGGCACCCGCAATCCTGCTAACAATGCTGCAATCGTG<br>CTACAACTTCCTCAAGGAACAACATTGCCAAAAGGCTTCTACG<br>CAGAAGGGAGCAGAGGCGGCAGTCAAGCCTCTTCTCGTTCCT<br>CATCACGTAGTCGCAACAGTTCAAGAAATTCAACTCCAGGCAG<br>CAGTAGGGGAACTTCTCCTGCTAGAATGGCTGGCAATGGCGGT<br>GATGCTGCTCTTGCTTTGCTGCTGCTTGACAGATTGAACCAGCT<br>TGAGAGCAAAATGTCTGGTAAAGGCCAACAACAACAAGGCCA<br>AACTGTCACTAAGAAATCTGCTGCTGAGGCTTCTAAGAAGCCT<br>CGGCAAAAACGTACTGCCACTAAAGCATACAATGTAACACAAG<br>CTTTCGGCAGACGTGGTCCAGAACAAACCCAAGGAAATTTTG<br>GGGACCAGGAACTAATCAGACAAGGAACTGATTACAAACATT<br>GGCCGCAAATTGCACAATTTGCCCCCAGCGCTTCAGCGTTCTT<br>CGGAATGTCGCGCATTGGCATGGAAGTCACACCTTCGGGAACG<br>TGGTTGACCTACACAGGTGCCATCAAATTGGATGACAAAGATC<br>CAAATTTCAAAGATCAAGTCATTTTGCTGAATAAGCATATTGAC<br>GCATACAAAACATTCCCACCAACAGAGCCTAAAAAGGACAAA<br>AAGAAGAAGGCTGATGAAACTCAAGCCTTACCGCAGAGACAG<br>AAGAAACAGCAAACTGTGACTCTTCTTCCTGCTGCAGATTTGG<br>ATGATTTCTCCAAACAATTGCAACAATCCATGAGCAGTGCTGA<br>CTCAACTCAGGCCTAA
B646L (VP72) gene of ASFV (accession: MN715134.1)	AAGGTTCACGTTCTCGTTAAACCAAAAGCGCAGCTTAATCCAG<br>AGCGCAAGAGGGGGCTGATAGTATTTAGGGGTTTGAGGTCCAT<br>TACAGCTGTAATGAACATTACGTCTTATGTCCAGATACGTTGCG<br>TCCGTGATAGGAGTGATATCTTGTTTACCTGCTGTTTGGATATTG<br>TGAGAGTTCTCGGGAAAATGTTGTGAAAGAAATTTCGGGTTGG<br>TATGGCTGCACGTTCGCTGCGTATCATTTTCATCGGTAAG

References

1. Sikorsky, J. A., Primerano, D. A., Fenger, T. W. & Denvir, J. DNA damage reduces Taq DNA polymerase fidelity and PCR amplification efficiency. *Biochem. Bioph. Res. Co.* **355**, 431–437 (2007).
2. Sikorsky, J. A., Primerano, D. A., Fenger, T. W. & Denvir, J. Effect of DNA damage on PCR amplification efficiency with the relative threshold cycle method. *Biochem. Bioph. Res. Co.* **323**, 823–830 (2004).
3. Epe, B., Ballmaier, D., Roussyn, I., Briviba, K. & Sies, H. DNA damage by peroxynitrite characterized with DNA repair enzymes. *Nucleic Acids Res.* **24**, 4105–4110 (1996).
4. Martínez, L., Andrade, R., Birgin, E. G. & Martínez, J. M. PACKMOL: a package for building initial configurations for molecular dynamics simulations. *J. Comput. Chem.* **30**, 2157–2164 (2009).
5. Ivani, I. et al. Parmbsc1: a refined force field for DNA simulations. *Nat. Methods* **13**, 55–58 (2016).
6. Heinz, H., Vaia, R. A., Farmer, B. L. & Naik, R. R. Accurate simulation of surfaces and interfaces of face-centered cubic metals using 12−6 and 9−6 Lennard-Jones potentials. *J. Phys. Chem. C* **112**, 17281–17290 (2008).
7. Case, D. A. et al. AMBER 2020. *University of California: San Francisco, CA, USA*, (2020).
8. Darden, T., York, D. & Pedersen L. Particle mesh Ewald: An N·log (N) method for Ewald sums in large systems. *J. Chem. Phys.* **98**, 10089–10092 (1993).
9. Swanson, J. M., Henchman, R.H. & McCammon, J. A. Revisiting free energy calculations: a theoretical connection to MM/PBSA and direct calculation of the association free energy. *Biophys. J.* **86**, 67–74 (2004).
10. Srinivasan, J., Cheatham, T. E., Cieplak, P., Kollman, P. A. & Case, D. A. Continuum solvent studies of the stability of DNA, RNA, and phosphoramidate-DNA helices. *J. Am. Chem. Soc.* **120**, 9401–9409 (1998).
11. Gilson, M. K. & Honig, B. Calculation of the total electrostatic energy of a macromolecular system: solvation energies, binding energies, and conformational analysis. *Proteins* **4**, 7–18 (1988).
12. Wang, J. M., Hou, T. J. & Xu, X. J. Recent advances in free energy calculations with a combination of molecular mechanics and continuum models. *Curr. Comput. Aid. Drug* **2**, 287–

306 (2006).

13. Miller III, B. R. et al. MMPBSA.py: an efficient program for end-state free energy calculations. *J. Chem. Theory Comput.* **8**, 3314–3321 (2012).
