# Supplementary material for: Fast microwave heating-based one-step synthesis of DNA and RNA modified gold nanoparticles
Source: Nat Commun. 2022 Feb 18;13:968. doi: 10.1038/s41467-022-28627-8 (PMC8857241; doi:10.1038/s41467-022-28627-8)
Supplement: Supplementary file 2 — Description of Additional Supplementary Files [file 41467_2022_28627_MOESM2_ESM.docx]

**Description of Additional Supplementary Files**

**Supplementary Movie 1:** The labeling process for DNA/RNA-AuNP conjugates via the MWassisted heating-dry method
