# Supplementary material for: Fast microwave heating-based one-step synthesis of DNA and RNA modified gold nanoparticles
Source: Nat Commun. 2022 Feb 18;13:968. doi: 10.1038/s41467-022-28627-8 (PMC8857241; doi:10.1038/s41467-022-28627-8)
Supplement: Supplementary file 4 — Source Data [file 41467_2022_28627_MOESM4_ESM.pdf]

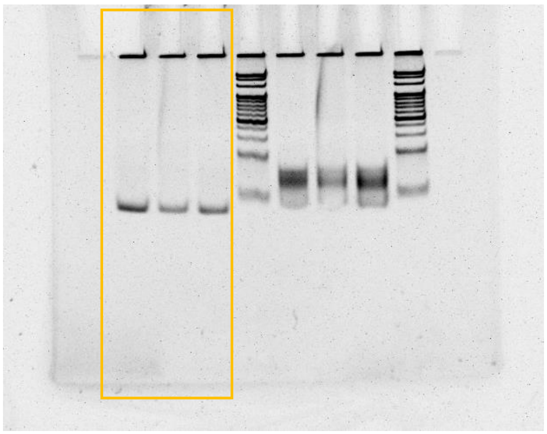

**Figure 1j.** Evaluation of the integrality of DNA strands by polyacrylamide gel electrophoresis.

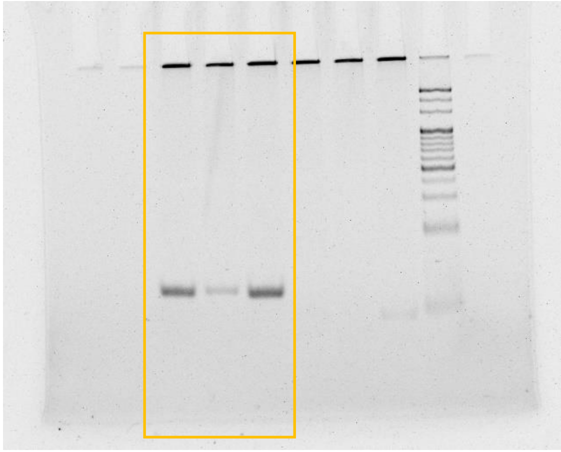

**Figure 1j.** Evaluation of the integrality of RNA strands by polyacrylamide gel electrophoresis.

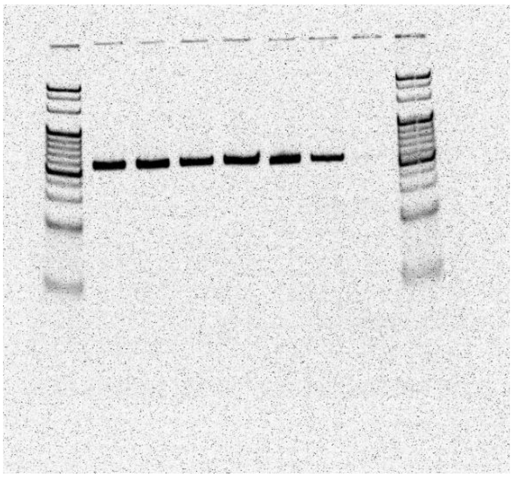

**Supplementary Figure 7b.** Gel electrophoresis analysis of PCR products amplified by different concentrations of DNA strand with or without MW-assisted heating-dry treatment.

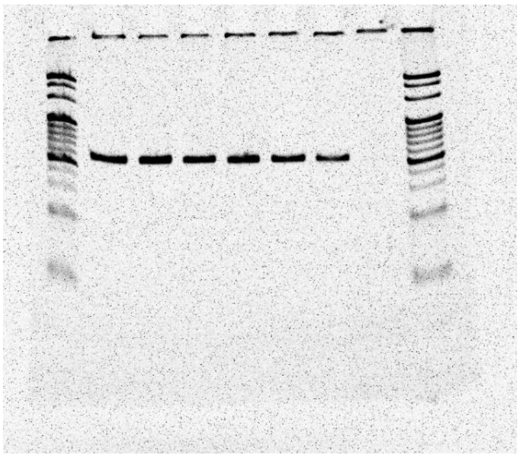

**Supplementary Figure 7b.** Gel electrophoresis analysis of PCR products amplified by different concentrations of DNA strand with or without MW-assisted heating-dry treatment.

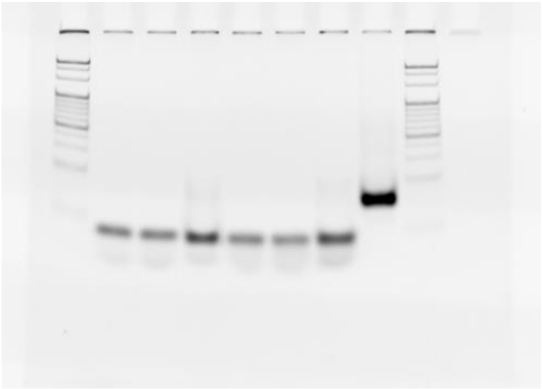

**Supplementary Figure 8b.** Gel electrophoresis analysis of short-chain ssDNA with or without MW-assisted heating-dry treatment in the absence/present of endonuclease.

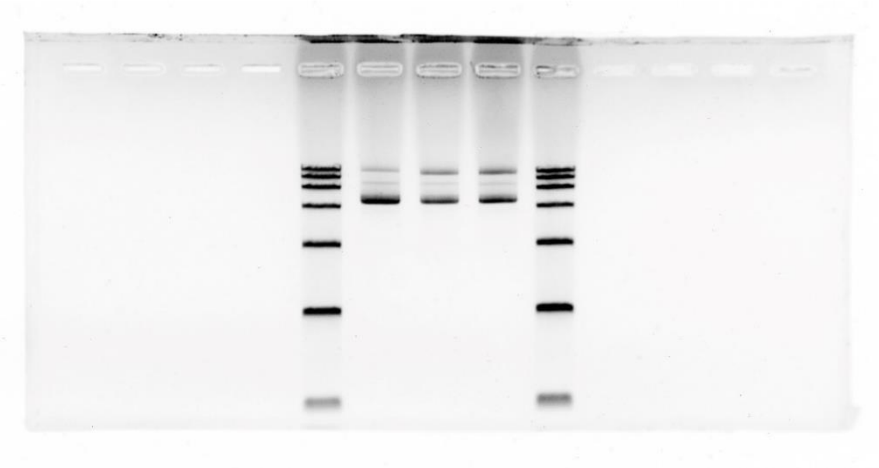

**Supplementary Figure 8b.** Gel electrophoresis analysis of long-chain EGFP plasmid DNA with or without MW-assisted heating-dry treatment in the absence/present of endonuclease.

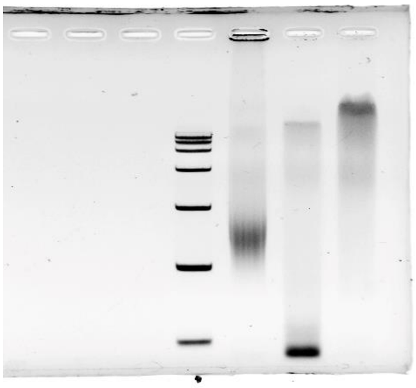

**Figure 6e.** Gel electrophoresis analysis of RCA products with perfectly matched or single-base mismatched target DNA.

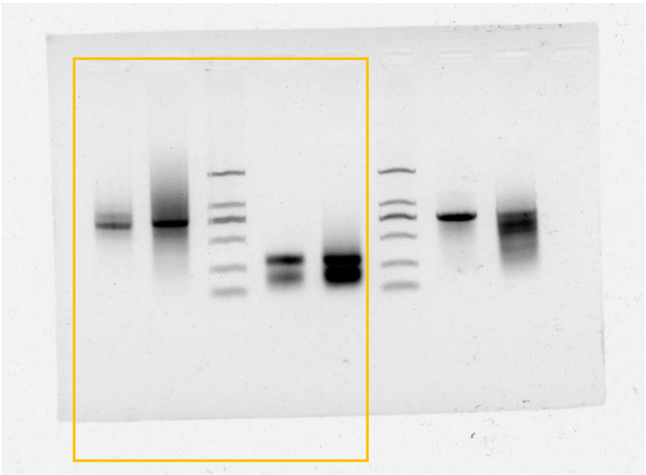

**Figure 6l.** Gel electrophoresis analysis of the in vitro transcribed sgRNA and N-RNA, and sgRNA and N-RNA displaced from AuNPs surface by ME.

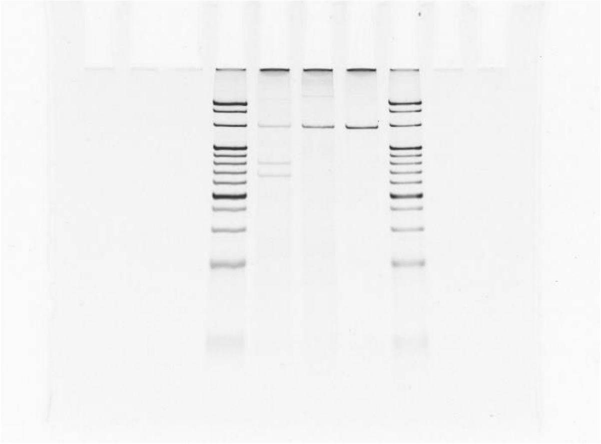

**Figure 6m.** Electrophoresis analysis of the cleavage ability of the assembled Cas9/sgRNA-AuNP complex.

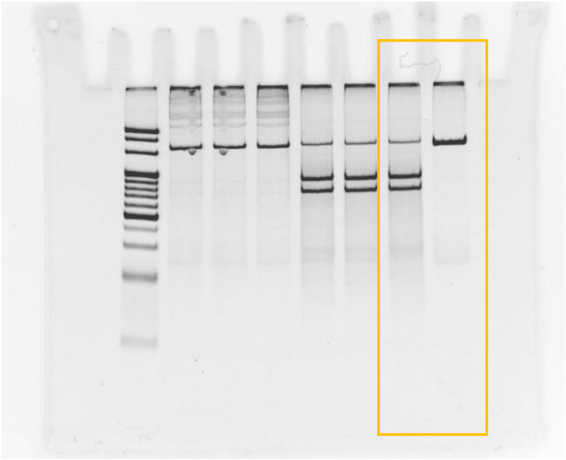

**Supplementary Figure 19a. Gel electrophoretic analysis.** Cleavage ability evaluation using designed poly (U)-tagged sgRNA and Cas9 protein.

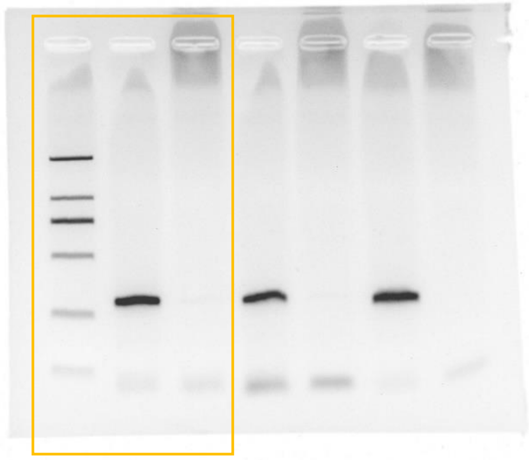

**Supplementary Figure 19b. Gel electrophoretic analysis.** Gel electrophoretic analysis of the PCR products of *B646L* (VP72) gene.
